# Teleost‐specific ictacalcins exhibit similar structural organization, cation‐dependent activation, and transcriptional regulation as human S100 proteins

**DOI:** 10.1111/febs.70354

**Published:** 2025-11-28

**Authors:** Liz Hernández, Théo Paris, Maria Demou, Catherine Birck, Christina Begon‐Pescia, Juan Francisco Rodríguez Vidal, Sylwia D. Tyrkalska, Charlotte Bureau, Catherine Gonzalez, Juliette Gracia, Etienne Lelièvre, Victoriano Mulero, Mai Nguyen‐Chi, Laure Yatime

**Affiliations:** ^1^ Laboratory of Pathogens and Host Immunity (LPHI) University of Montpellier, INSERM UA15, CNRS UMR5294 France; ^2^ Integrated Structural Biology Platform, CBI‐IGBMC, CNRS UMR 7104, INSERM U1258 University of Strasbourg Illkirch France; ^3^ Departamento de Biología Celular e Histología, Facultad de Biología Universidad de Murcia Spain; ^4^ Instituto Murciano de Investigación Biosanitaria (IMIB) Pascual Parrilla Murcia Spain; ^5^ Centro de Investigación Biomédica en Red de Enfermedades Raras (CIBERER) Instituto de Salud Carlos III Madrid Spain

**Keywords:** cation binding, inflammatory models, S100 proteins, X‐ray crystallography, zebrafish

## Abstract

S100 proteins are highly versatile calcium‐binding proteins from vertebrates. Following extracellular release, they become essential in immune and antimicrobial defenses, initiating the inflammatory response through receptor signaling and providing direct control of bacterial invaders via nutritional immunity. While mammalian S100s have been extensively studied, very little is known about the more recently discovered S100 proteins from teleost fish, including those with no strict orthologs in mammals. Comparable functioning between both clades would allow us to expand their study into the highly popular zebrafish model, which is particularly suited for live imaging and mechanistic exploration of immune and inflammatory processes. To fill the gap of knowledge on teleost S100s, we here provide detailed structural and biochemical characterization of S100i1 and S100i2 from *Danio rerio*, two teleost‐specific S100s absent in mammals. We demonstrate that they nevertheless share conserved tertiary and quaternary organization with mammalian S100s. In addition, they exhibit comparable calcium binding properties and undergo a similar calcium‐dependent activation mechanism. Furthermore, they display analogous expression patterns, being enriched in tissues highly exposed to the environment such as gills and skin, the latter constituting an important reservoir of S100 proteins in mammals. Finally, our results show, for the very first time, that *s100i2*/*i2* gene expression is differentially modulated in sterile disease conditions associated with sustained inflammation or a high hypoxic state. Altogether, these findings underline the strong parallelism existing between mammalian and teleost‐specific S100 proteins despite their divergent evolution, opening up new avenues to explore their biology in the zebrafish model.

## Graphical abstract

Calcium‐binding S100 proteins are important mediators of inflammation in humans. Distant members of this family have been recently discovered in teleost fish but their resemblance to human proteins has not been explored yet. This study demonstrates that zebrafish‐specific S100i1 and S100i2 (ictacalcins) possess identical structural organization and calcium‐dependent activation as their human counterparts and are likewise transcriptionally upregulated in chronic disease contexts associated with sustained inflammation or hypoxia.

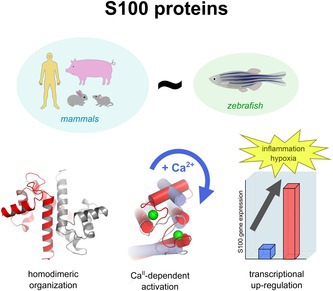

**Abbreviations**
ADP: atomic displacement parameters
dpf: days post fertilization
eng: endoglin
HHT: hereditary hemorrhagic telangiectasia
hpa: hours post amputation
hpf: hours post fertilization
Il‐1b: interleukin 1b
ITC: isothermal titration calorimetry
mfap4: microfibril‐associated protein 4
mpx: myeloperoxidase
MR: molecular replacement
Ni‐NTA: Nickel‐affinity chromatography
rag1: recombination activating gene 1
RAGE: receptor for advanced glycation end‐products
rmsd: root mean square deviation
SEC: size exclusion chromatography
Spint1a: serine peptidase inhibitor, Kunitz type 1a
TLS: translation/libration/screw
tnf: tumor necrosis factor

## Introduction

S100 proteins are small proteins of 10–15 kDa that form the largest subgroup within the EF‐hand superfamily of calcium‐binding proteins. They are exclusively found in vertebrates and exert pleiotropic functions, both inside and outside cells, acting as calcium‐dependent regulators of many vital processes, such as metabolism, cell proliferation, and cytoskeleton rearrangement within cells, or immune and antibacterial defenses in the extracellular milieu [1, 2]. Noteworthily, several S100 members are considered major effectors of innate immunity in humans. In response to infections or sterile injuries, they are released into the extracellular environment, where they contribute both to the propagation of inflammation, through their alarmin function, and to the antimicrobial response, through nutritional immunity [3, 4]. Their role in inflammation is, however, double‐edged, as long‐lasting injuries allow for strong upregulation of their gene expression, thus promoting their continuous release. This feeds the inflammatory response up to a nonresolving state that leads to aggravated damage and, ultimately, to chronic pathologies [5]. Hence, master regulators of inflammation such as the S100A8/A9 heterodimer are considered both valuable biomarkers and promising therapeutic targets in numerous disease conditions linked to aberrant inflammation [6].

Given the importance of S100 proteins in human physiopathology, animal models to explore their function in health and disease have long been developed, predominantly in rodents. Although early evidence suggested they may be widespread within vertebrates [7], S100 proteins have to date mostly been studied in mammals, in which around 20 members are commonly annotated. Recent phylogenetic analyses allowed for the characterization of *S100* orthologous genes in distinct vertebrate clades, including amphibians and teleost fish [8]. Among the latter, zebrafish has become a highly popular animal model to dissect immune and inflammatory processes, having a high degree of genetic and immune conservation with mammals and offering selective advantages as compared to mice models, notably its transparency at larval stages that allows for following physiological processes in real time thanks to intravital imaging [9, 10]. Despite such attractive traits, zebrafish has so far not been considered extensively for the study of S100‐related processes, and in fact, still very little is known about the expression and function of zebrafish S100 family members [11].

Fourteen *s100* genes have been identified in *Danio rerio*, only six of which are conserved with mammals [8]. Interestingly, the eight other *s100* genes from zebrafish, and from teleost fish in general, do not share any evident phylogenetic lineage with mammalian *S100* genes, which questions the conservation of their gene products in terms of structural and functional features [8, 12]. The first teleost‐specific gene was discovered in the chemosensory tissues of catfish (*Ictalurus punctatus*) and was therefore termed ictacalcin (*icn*) [13]. Two paralogs of this gene, which arose from the third whole genome duplication event, were more recently identified in zebrafish: *icn* and *icn2*, also referred to as *s100i1* and *s100i2* for consistency with the S100 family nomenclature [14]. While these two fish *s100* genes have been studied in slightly more detail due to their predominant expression in epithelial tissues, the degree of resemblance of their products with their mammalian counterparts, and thus the possible parallelism in biological functions, remains to be clarified.

To gain insights into how zebrafish‐specific S100 proteins compare to mammalian ones, we here provide a detailed structural and biochemical characterization of the two ictacalcin proteins from *Danio rerio*, combining X‐ray crystallography and isothermal titration calorimetry (ITC). Using the zebrafish model, we further explore ictacalcin gene expression under homeostatic conditions and in various sterile disease models, revealing *S100i1/i2* transcriptional modulation in conditions associated with sustained inflammation or high hypoxic states. Altogether, these findings highlight the strong similarities existing between mammalian and teleost‐specific S100 proteins, hinting at possible analogous functions despite their divergent evolution.

## Results

### Zebrafish ictacalcins possess conserved tertiary and quaternary organization with mammalian S100 proteins

**Fig. 1:**
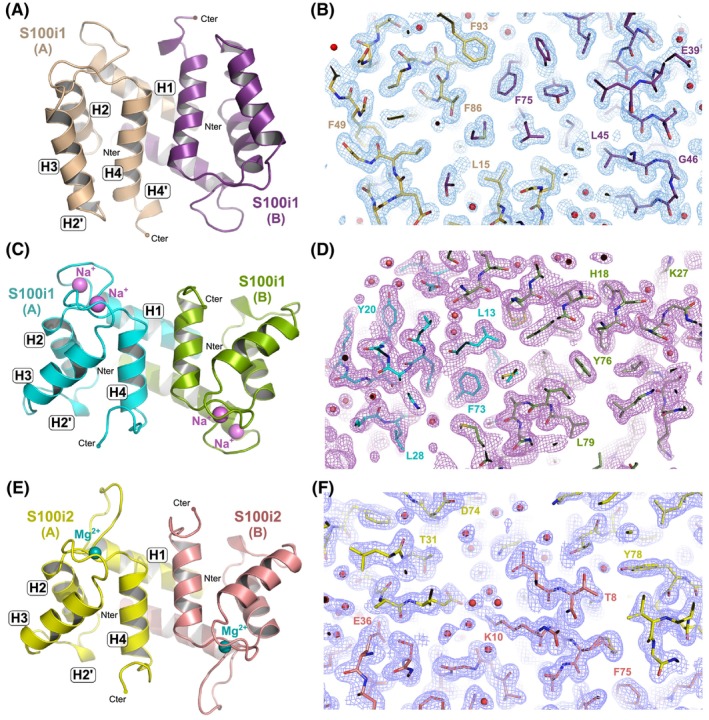
Crystallographic structures of zebrafish ictacalcins in various ionic states. (A) General overview of the structure of apo S100i1 homodimer at 1.5 Å resolution. The two protomers A and B are shown in beige and purple. (B) Final electron density map contoured at 1σ (blue mesh) with final model superimposed. (C) General overview of the structure of Na^+^‐bound S100i1 homodimer at 1.65 Å resolution. The two protomers A and B are shown in cyan and olive. Sodium ions are displayed as magenta spheres. (D) Final electron density map contoured at 1σ (purple mesh) with final model superimposed. (E) General overview of the structure of Mg^2+^‐bound S100i2 homodimer at 1.6 Å resolution. The two protomers A and B are shown in yellow and salmon. Magnesium ions are displayed as blue spheres. (F) Final electron density map contoured at 1σ (purple blue mesh) with final model superimposed. For each structure, helix nomenclature is indicated on protomer A. The four main helices are labeled as H1 to H4, and the two small helix turns are indicated as H2' and H4'. Structural models and electron density maps were drawn with pymol Molecular Graphics System (version 215 0.99rc6, DeLano Scientific LLC).

At the protein sequence level, S100i1 and S100i2 exhibit the strongest homology with human S100A4 (53.2% and 50.0% sequence identity, respectively) and human S100A2 (50.0% and 47.9% sequence identity, respectively). However, it is unknown whether they share all structural features of the human proteins. To assess the structural properties of zebrafish ictacalcins in detail, we produced them as recombinant proteins and crystallized them in various ionic conditions, yielding three distinct crystal forms that all diffracted to fairly high resolution (Table 1) and which we attributed to apo S100i1 (crystal form 1), Na^+^‐bound S100i1 (crystal form 2) and Mg^2+^‐bound S100i2 (see later). The final atomic models, covering residues Ala2 to Thr92/Gly93 for S100i1 and residues Met5 to Phe92 for S100i2, are displayed in Fig. 1, together with final electron density maps. At the protomer level, both S100i1 and S100i2 adopt the classical four‐helix bundle fold characteristic of S100 proteins (Fig. 1A–F). The four helices are formed by the same stretches of residues in both proteins, corresponding in S100i1 to Ser4‐Ser21 (helix H1), Ser31‐Leu43 (helix H2), Asp52‐Asp64 (helix H3) and Asp72‐Leu86 (helix H4). A short α‐helix turn (H2′) is also observed both in S100i1 and S100i2, in the hinge region linking helices H2 and H3, that covers residues Gly44 to Phe47 (S100i1 numbering), and another short α‐helix turn (H4′) is present after helix H4 in the apo S100i1 structure, encompassing residues Glu89 to Thr92. The last five C‐terminal residues are not visible in the density, most probably corresponding to flexible regions without defined secondary structure.

**Table 1:** Data collection and refinement statistics. Values indicated in parentheses correspond to the last shell of resolution.

	Apo S100i1 (crystal form 1)	Na^+^‐S100i1 (crystal form 2)	Mg^2+^‐S100i2
Data collection			
Diffraction source	X06DA, SLS	X06DA, SLS	X06DA, SLS
Wavelength (Å)	1.000	1.000	1.000
Space group	P2_1_	P2_1_	P2
*a*, *b*, *c* (Å)	34.38, 131.01, 45.82	33.23, 55.86, 45.02	32.58, 58.28, 41.66
α, β, γ (°)	90, 90.089, 90	90, 99.998, 90	90, 91.59, 90
Mosaicity (°)	0.087	0.09	0.20
Resolution range (Å)	50–1.5 (1.57–1.5)	50–1.65 (1.71–1.65)	50–1.6 (1.67–1.6)
Total No. of reflections	421 231 (45 235)	131 795 (13 159)	136 922 (15 560)
No. of unique reflections	61 980 (7421)	19 587 (1990)	20 615 (2473)
Completeness (%)	96.0 (90.3)	99.8 (99.6)	100 (99.9)
Redundancy	6.8 (6.1)	6.7 (6.6)	6.6 (6.3)
*I*/σ(*I*)	22.85 (2.59)	21.33 (1.57)	20.60 (1.73)
*R*_meas_	5.4 (75.0)	4.2 (120.2)	4.2 (121.7)
Wilson B factor (Å^2^)	23.1	33.2	33.3
Refinement			
Resolution range (Å)	50–1.5	50–1.65	50–1.6
No. of unique reflections	61 633	19 253	20 341
Final *R*_work_/*R*_free_ (%)	16.81/20.03	20.93/23.19	22.42/24.35
No. of non‐H atoms			
Protein	2917	1398	1395
Ions	0	4	2
Water	492	89	96
Total	3409	1491	1493
R.m.s. deviations			
Bonds (Å)	0.010	0.005	0.003
Angles (°)	1.039	0.751	0.648
Average B factors (Å^2^)			
Protein	26.4	65.9	51.1
Ions	–	25.6	19.1
Water	37.3	46.3	49.3
Ramachandran plot			
Favored regions (%)	98.9	96.0	94.2
Allowed regions (%)	1.1	4.0	5.8
Outliers (%)	0	0	0
PDB code	9I2N	9HYG	9I1G

**Fig. 2:**
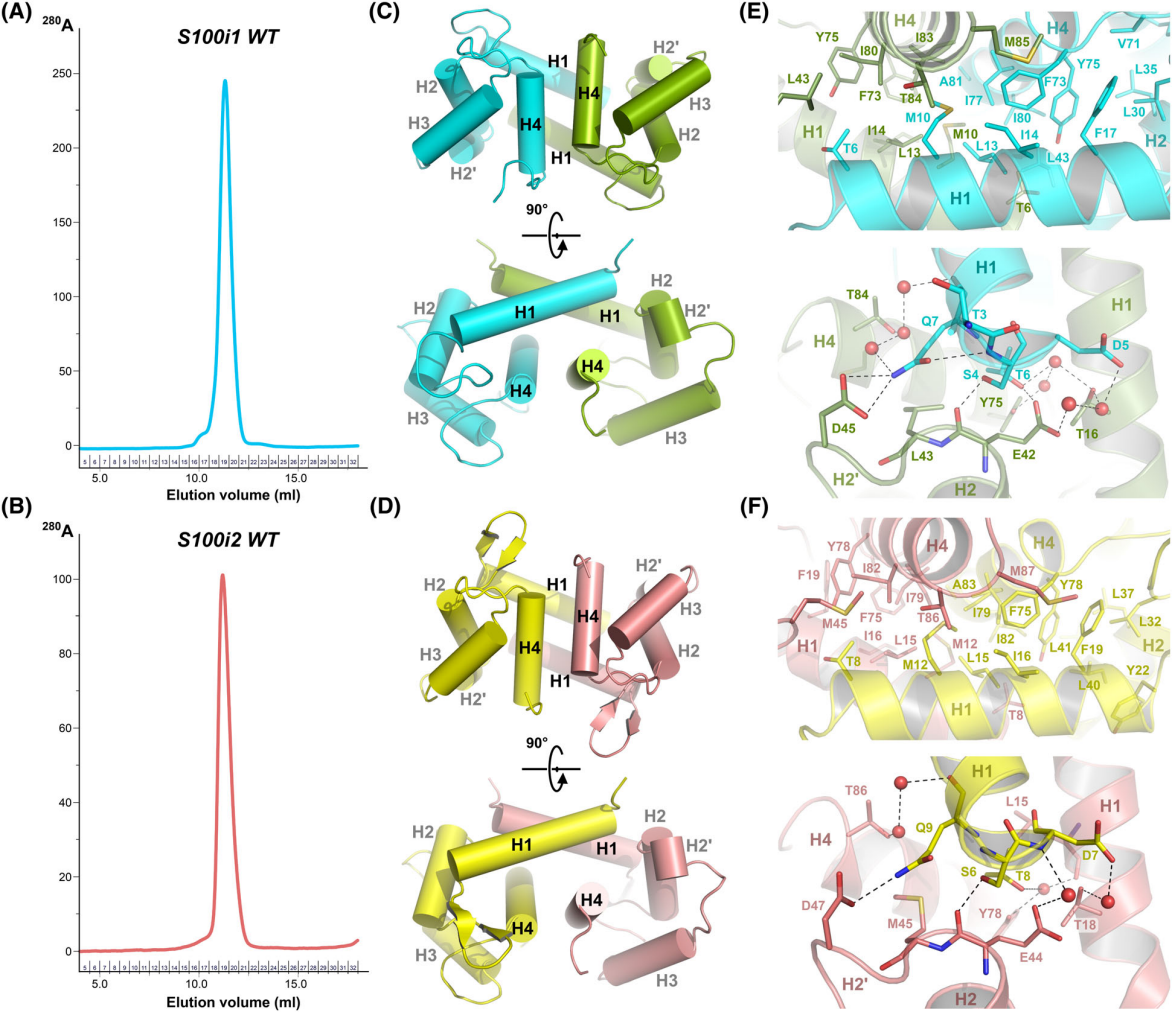
Homodimeric organization of zebrafish ictacalcins. (A, B) Representative Size Exclusion Chromatography (SEC) elution profiles of WT S100i1 (A) and WT S100i2 (B) on Superdex 75 Increase, revealing predominant elution as a homodimer. SEC experiments were repeated thrice (*n* = 3). (C) Helix packing in the S100i1 homodimer. The two protomers A and B are shown in cyan and olive. The four main helices are labeled as H1 to H4, and the small helix turn between H2 and H3 is indicated as H2'. (D) Helix packing in the S100i2 homodimer. The two protomers A and B are shown in yellow and salmon. The four main helices are labeled as H1 to H4, and the small helix turn between H2 and H3 is indicated as H2'. (E) Hydrophobic (upper panel) and electrostatic (lower panel) interactions stabilizing the S100i1 dimer interface. (F) Hydrophobic (upper panel) and electrostatic (lower panel) interactions stabilizing the S100i2 dimer interface. Structural models were drawn with pymol Molecular Graphics System (version 215 0.99rc6, DeLano Scientific LLC).

As expected for S100 proteins, and in agreement with their elution profile on size exclusion chromatography (SEC; Fig. 2A,B), both ictacalcins organize into homodimeric assemblies that follow the canonical H1–H1–H4–H4 packing seen in most S100 structures (Fig. 2C,D). In both proteins, the dimer interface is held in place by an extended network of hydrophobic interactions involving the side chains of aromatic and aliphatic residues carried by the central portions of H1 and H4 from both protomers (Fig. 2E,F, upper panels). The interface is further stabilized at both extremities by a smaller network of hydrogen bonds involving side chains of polar residues at the N terminus of H1 from one protomer, and at the hinge region (H2–H3 linker) and C terminus of H4 from the second protomer, either through direct contacts such as Ser4‐Glu42, Thr6‐Glu42, or Gln7‐Asp45 (S100i1 numbering), or through water‐mediated inter‐protomer interactions (Fig. 2E,F, lower panels). The interface areas calculated by PISA [15] cover 1105 Å^2^ for S100i1 and 814 Å^2^ for S100i2, with 34 and 37 interfacing residues, respectively, and 11 hydrogen bonds in both cases. The overall solvation free energy gain is of −21.4 and −15.4 kcal·mol^−1^ for s100i1 and s100i2 dimers, respectively, with associated *P*‐values of 0.114 and 0.043, which highlights the strong hydrophobicity and stability of the interfaces. Altogether, this demonstrates that zebrafish ictacalcins adopt a quaternary organization identical to that of mammalian S100s.

### Ictacalcins display conserved cation binding mode in their EF‐hand motifs

**Fig. 3:**
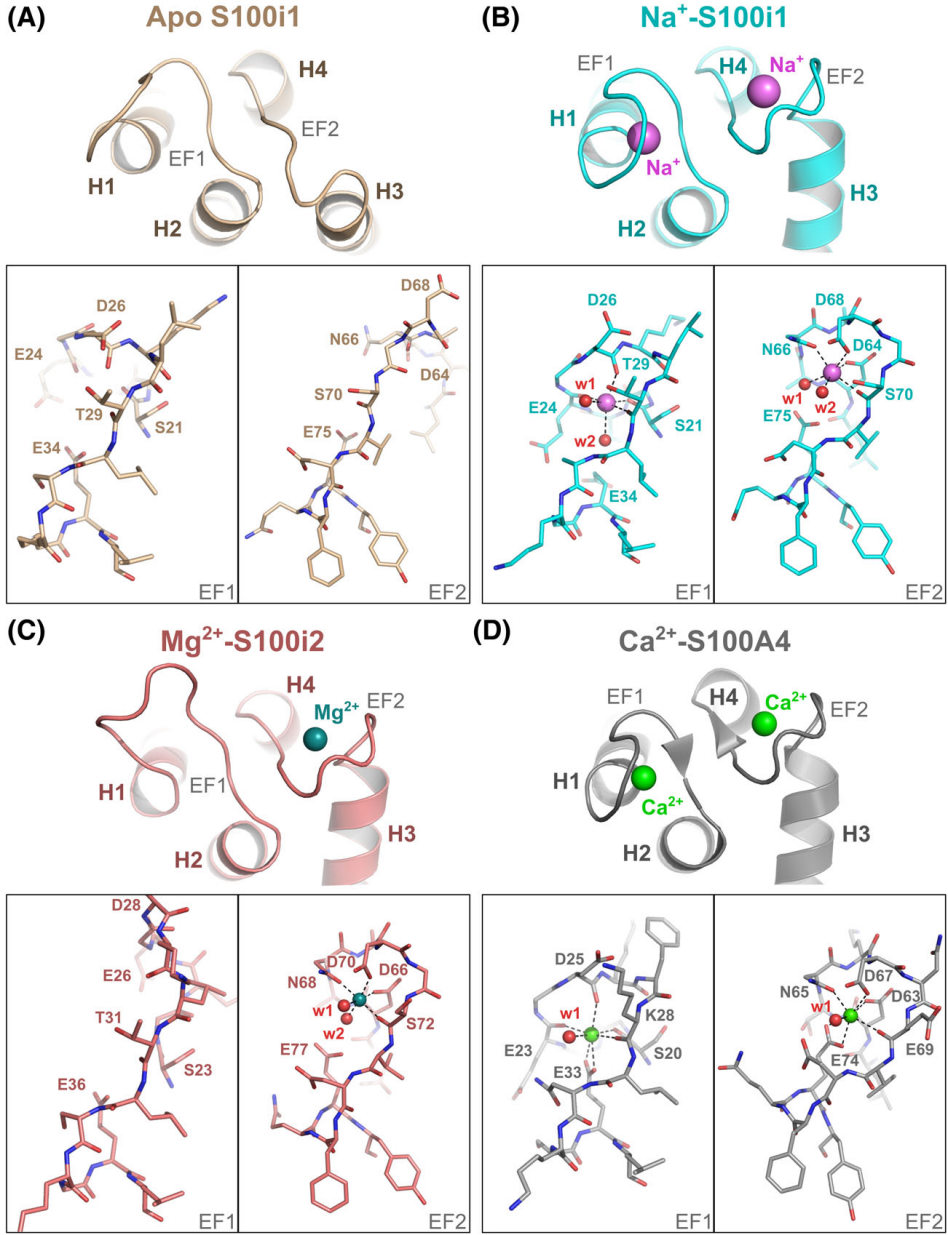
Cation binding in the EF‐loops of S100i1 and S100i2. (A–C) Close‐up view on the overall conformation and cation binding residues within the EF‐loops of apo S100i1 (A), Na^+^‐bound S100i1 (B) and Mg^2+^‐bound S100i2 (C). (D) Comparison with the EF‐loops of Ca^2+^‐bound human S100A4 (PDB_ID 2Q91 [16]). Sodium, magnesium and calcium ions are shown as magenta, blue and green spheres, respectively. Structural models were drawn with pymol Molecular Graphics System (version 215 0.99rc6, DeLano Scientific LLC).

The presence of various cations in the crystallization buffers allowed trapping S100i1 and S100i2 in several ionic states. Hence, while the EF‐loops of S100i1 in crystal form 1 are devoid of cations (Fig. 3A), two Na^+^ ions are found in each S100i1 protomer of crystal form 2, occupying both EF‐loops of the protein (Fig. 3B). Additionally, one Mg^2+^ ion is found in the second EF‐loop (EF2; H3–H4 linker) of each S100i2 protomer, whereas the first EF‐loop (EF1; H1–H2 linker) remains empty (Fig. 3C). Comparison of the EF‐loop conformations in both apo and ion‐bound states reveals important conformational rearrangements, as for mammalian S100s. The two EF‐loops shift from a rather extended conformation when empty, to a more compact architecture when occupied, wrapping around the central ion as classically observed in Ca^2+^‐loaded human S100 proteins (Fig. 3D).

These structures also reveal the residues involved in cation binding in both EF‐hand motifs, the average ion‐oxygen distances being of 2.43 Å for Na–O bonds and 2.06 Å for Mg–O bonds, in agreement with mean values reported in the literature [17, 18]. Sodium displays an octahedral coordination sphere in both EF1 and EF2, with hydrogen bonds provided by main chain carbonyls of Ser21, Glu24, Asp26, Thr29 (EF1), and Ser70 (EF2), side chain oxygens of Asp64, Asn66, and Asp68 (EF2) and two water molecules in each EF‐loop (held in place by Glu75 in EF2). Magnesium has similar octahedral coordination geometry in EF2 of S100i2, with the same residues implicated in cation chelation, namely Asp66, Asn68 and Asp70 through side chain oxygens, Ser72 through main chain carbonyl and two water molecules, brought by Glu77. Like in mammals, the two S100i1/i2 EF‐loops are thus non‐equivalent, the second one providing coordination mainly via side chain oxygens, thus corresponding to a canonical EF‐loop, whereas the first loop chelates cations via main chain carbonyl groups, thereby corresponding to a pseudo EF‐loop (S100‐specific).

**Fig. 4:**
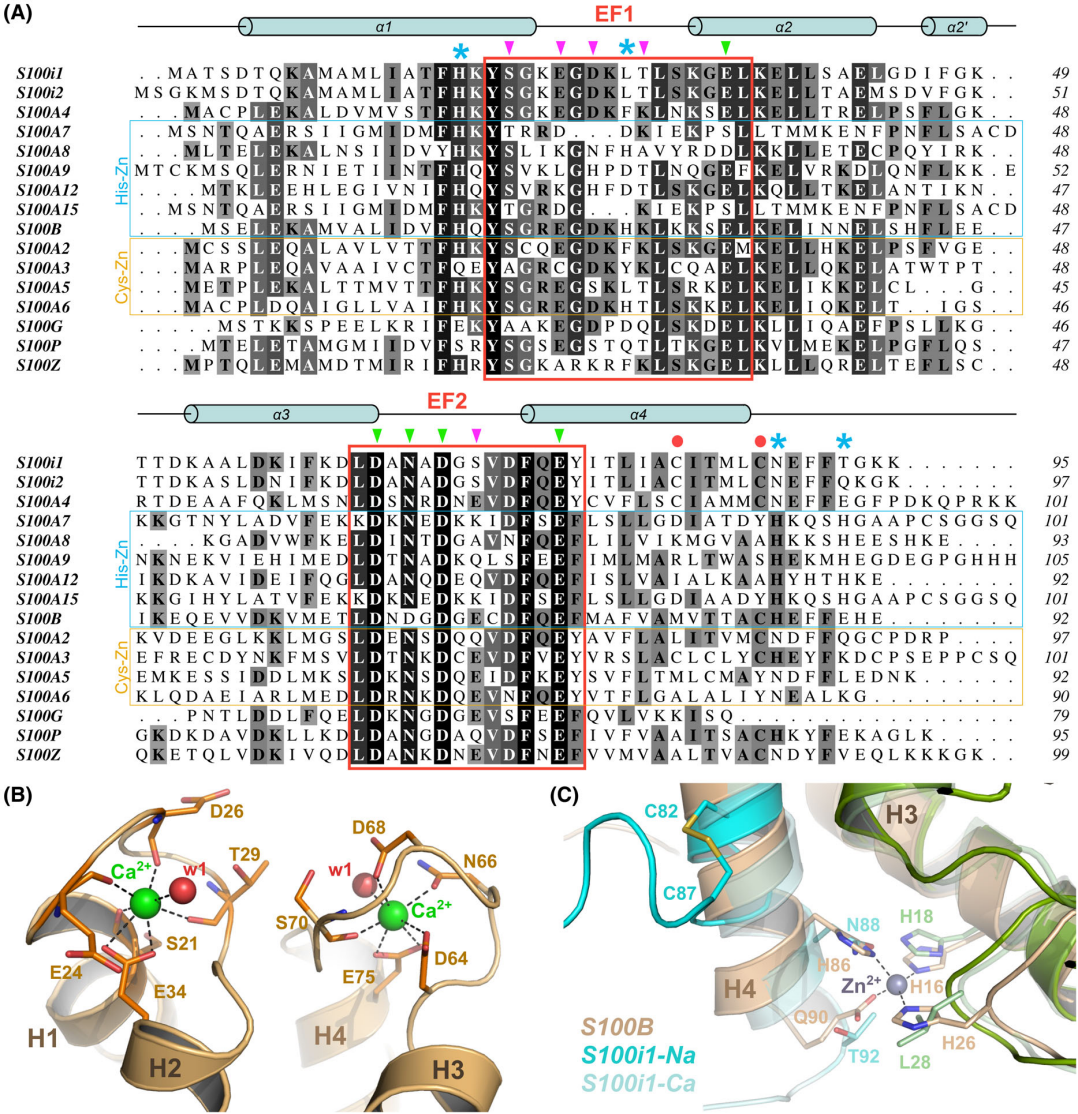
Conservation of the calcium binding mode between zebrafish S100i1/i2 and human S100s. (A) Sequence alignment of zebrafish S100i1/i2 with human S100s, highlighting the high degree of sequence conservation, especially in the EF‐loop motifs (indicated by the red boxes). Residues from EF‐loops involved in calcium chelation through main chain or side chain atoms are indicated on top of the alignment with purple or green arrows, respectively. Residues involved in zinc binding in the His‐Zn human S100s are indicated with blue asterisks. Cysteine residues involved in the intramolecular disulfide bridge in the second half of helix H4 in S100i1/i2 are indicated by a red dot. The sequences of human and zebrafish proteins are from the UniProt database with the following accession codes: Q6XG62 (zebrafish S100i1), A3FKT8 (zebrafish S100i2), P26447 (human S100A4), P31151 (human S100A7), P05109 (human S100A8), P06702 (human S100A9), P80511 (human S100A12), Q86SG5 (human S100A15), P04271 (human S100B), P29034 (human S100A2), P33764 (human S100A3), P33763 (human S100A5), P06703 (human S100A6), P29377 (human S100G), P25815 (human S100P), and Q8WXG8 (human S100Z). Sequence alignments were done in clustal omega [19] and coloring according to conservation was done in aline [20]. (B) alphafold‐predicted 3D model of Ca^2+^‐bound S100i1 homodimer highlighting a similar binding mode as human S100s. (C) Superimposition of Zn^2+^‐bound human S100B (beige and olive cartoons; PDB_ID 3D0Y [21]) with Na^+^‐ and predicted Ca^2+^‐bound S100i1 showing that the His‐Zn site of S100B is not conserved in S100i1. Instead, upon Na^+^ binding at least, the formation of an intramolecular disulfide bridge between Cys82 and Cys87 pushes the S100i1 C‐terminal tail away from the homodimer interface. Structural models were drawn with pymol Molecular Graphics System (version 215 0.99rc6, DeLano Scientific LLC).

Strong conservation of the S100i1/i2 residues involved in Na^+^/Mg^2+^ chelation with those involved in Ca^2+^ coordination in human S100s (Fig. 4A) allows us to postulate a conserved calcium binding mode, as illustrated with the 3D model of Ca^2+^‐bound S100i1/i2 homodimer (shown in Fig. 4B,C for S100i1) that we generated. In this model, the EF‐loop conformations and overall coordination spheres strongly resemble those observed in Na^+^‐S100i1 except that the lateral water molecule at the octahedron basis is now replaced by the two oxygen atoms of Glu34 in EF1 or Glu75 in EF2, thus giving rise to a heptahedral coordination sphere with pentagonal bipyramidal geometry, as observed in Ca^2+^‐bound human S100A4 (Fig. 3D).

### Cation binding properties of zebrafish ictacalcins

**Fig. 5:**
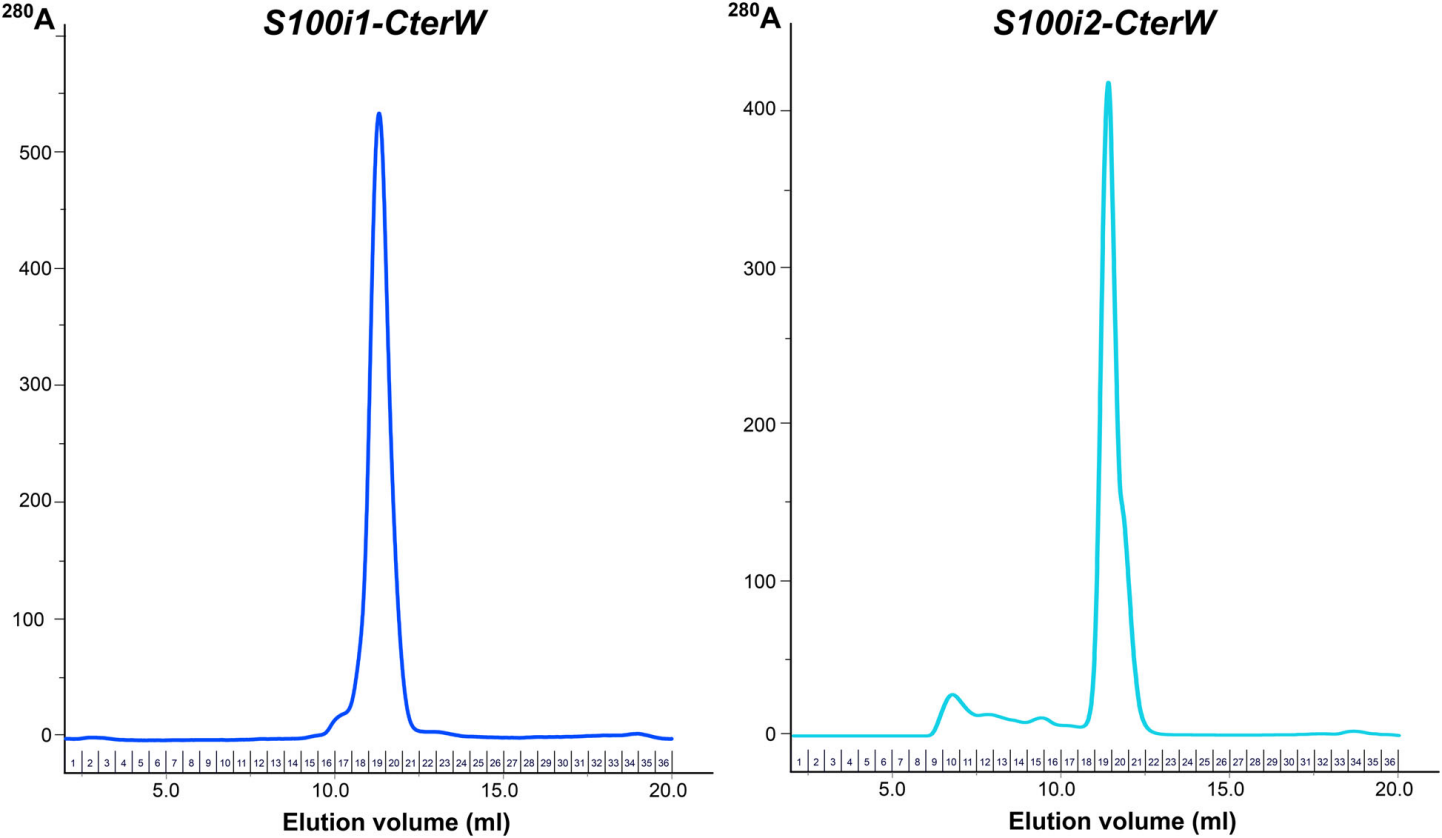
SEC elution profiles of the S100i1 and S100i2 CterW variants. Elutions were performed on a 24 mL Superdex 75 Increase column equilibrated in 20 mm Tris–HCl pH 7.5, 100 mm NaCl. For each protein, at least three independent elutions were performed (*n* = 3). Both proteins eluted predominantly as homodimers, as well as WT proteins.

**Fig. 6:**
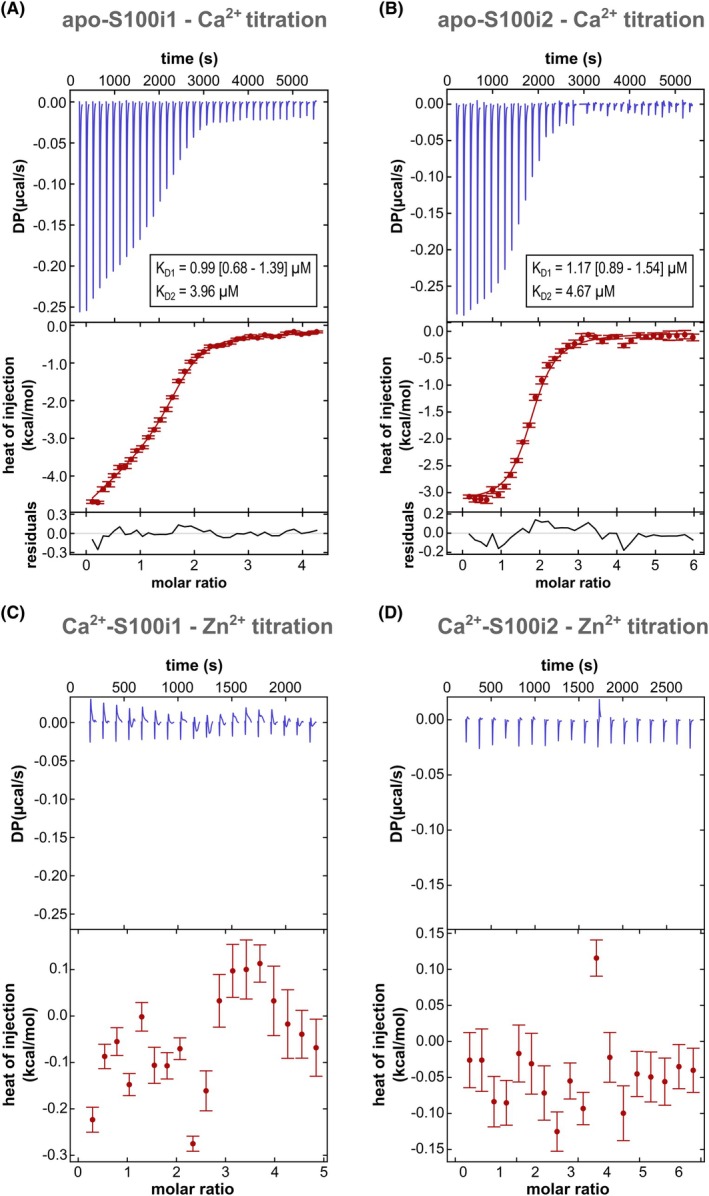
Cation binding properties of zebrafish S100i1/i2. (A, B) Representative Isothermal Titration Calorimetry (ITC) experiment for S100i1 (A) or S100i2 (B) binding to Ca^2+^. The upper graphs show reconstructed thermograms from nitpic software [22], the middle graphs show binding isotherms and global fit, and the bottom graphs show the fitting residuals. Error bars were calculated in nitpic and reflect the calculated error of integrated heats arising from peak shape analysis. Shown are titrations of 0.4 mm CaCl_2_ into 40 μm S100i1 and 0.6 mm CaCl_2_ into 42 μm S100i2. In the global analysis, a total of three independent titrations (*n* = 3) were included for S100i1 or S100i2 binding to Ca^2+^, respectively. Macroscopic dissociation constants (*K*_D_) obtained from global analysis with two symmetric sites per protomer are displayed with the 95% confidence interval indicated in brackets. (C, D) Representative ITC titrations of S100i1 (C) or S100i2 (D) binding to Zn^2+^. The upper graphs show reconstructed thermograms from nitpic and the bottom graphs show the corresponding heat changes. Error bars were calculated in nitpic and reflect the calculated error of integrated heats arising from peak shape analysis. Shown are titrations of 1.0 mm Zn acetate into 40 μm S100i1 and 0.6 mm Zn acetate into 60 μm S100i2. No binding to Zn^2+^ can be detected for any of the proteins.

To characterize the binding properties of the calcium sites, we performed ITC experiments on the two zebrafish ictacalcins. To ensure better accuracy in the determination of the corresponding dissociation constants (*K*_D_), we generated CterW variants encompassing an additional Trp residue at the end of the sequence, thus increasing the molar extinction coefficient for a more robust assessment of the protein concentration. Both variants gave similar production yields, displayed similar SEC elution profiles (i.e., homodimers; Fig. 5), and behaved identically to WT proteins. Despite the presence of two binding sites per protomer, a single discernible transition was observed in the ITC titration curves (Fig. 6A,B). Analysis with a macroscopic model of ‘two symmetric sites’ or a microscopic model of ‘two non‐symmetric sites’ gave almost similar results but the calculated parameters were better defined using the macroscopic model. The macroscopic *K*_D1_ and *K*_D2_ values are respectively 0.99 and 3.96 μm for S100i1 and 1.17 and 4.67 μm for S100i2 (Table 2). For S100i1, the microscopic *K*_D_ would be 1.98 μm (2 × *K*_D1_) for both Ca^2+^ binding sites, which is very close to the fitted *K*_D_ value of 1.99 [0.68–3.77] μm found for each site using the microscopic model. For S100i2, the microscopic *K*_D_ would be 2.34 μm for both Ca^2+^ binding sites compared to the fitted *K*_D_ values of 0.23 [0.003–5.68] μm and 4.07 [no limit found] μm for each site using the microscopic model. The enthalpy values are much less defined using this latter model and no cooperativity between the two sites was revealed. Hence, despite the nonequivalence of the two sites in terms of cation chelating mode, S100i1 and S100i2 bind to calcium with rather close affinities in the two EF‐loops, which is often observed for S100 proteins [23, 24].

**Table 2:** Dissociation constants and enthalpies for the binding of calcium to zebrafish ictacalcins as measured by ITC. Fitting was performed using a macroscopic model of ‘two symmetric sites’. Values in brackets indicate a 95% confidence interval [2σ] determined by error‐surface projection in the global analysis of three independent experiments (*n* = 3).

Protein	*K*_D1_ (μm)	Δ*H*_1_ (kcal·mol^−1^)	*K*_D2_ (μm)	Δ*H*_2_ (kcal·mol^−1^)	Incompetent fraction (%)
S100i1	0.99 [0.68 to 1.39]	−4.60 [−4.75 to −4.46]	3.96	−2.48 [−3.08 to −1.92]	0.15 [0.10 to 0.19]
S100i2	1.17 [0.89 to 1.54]	−3.14 [−3.25 to −3.04]	4.67	−3.14 [−3.69 to −2.96]	0.16 [0.13 to 0.19]

Several mammalian S100s, notably those involved in antimicrobial defenses through nutritional immunity, also bind transition metals, at sites distinct from the calcium EF‐hands [25]. To evaluate the metal binding capacity of S100i1/i2, we next performed zinc titrations in both the apo and Ca^2+^‐loaded proteins. No binding could be detected, for either protein (Fig. 6C,D). This corroborates the fact that only one of the four Zn^2+^‐chelating residues present in human S100s is conserved in S100i1/i2, the second His/Asp Zn‐ligand in EF1 being instead a leucine, and the two His ligands in the second half of H4 being replaced by an Asn and a Thr/Gln in S100i1/i2 (Fig. 4A). Therefore, as modeled in Fig. 4C in comparison with the Zn^2+^ site of human S100B, the canonical His‐Zn site at the interface between the two S100 protomers cannot form in S100i1/i2. Two Cys residues are instead present at the end of H4 in zebrafish ictacalcins but they do not serve as surrogates for metal chelation. Indeed, in our structural models, binding of Na^+^ or Mg^2+^ to S100i1/i2 triggers an unwinding of H4 by one helix turn and leads to the formation of an intramolecular disulfide bridge between these two cysteines (Cys82 and Cys87 in S100i1) that holds the C‐terminal tail away from the dimer interface, thus preventing favorable positioning of helix H4 C terminus for the formation of a metal binding site at the dimer interface. This S–S bridge would evidently only occur in extracellular S100i1/i2, providing they indeed get released outside cells—which still awaits experimental confirmation.

### Ictacalcins undergo cation‐dependent conformational rearrangements

**Fig. 7:**
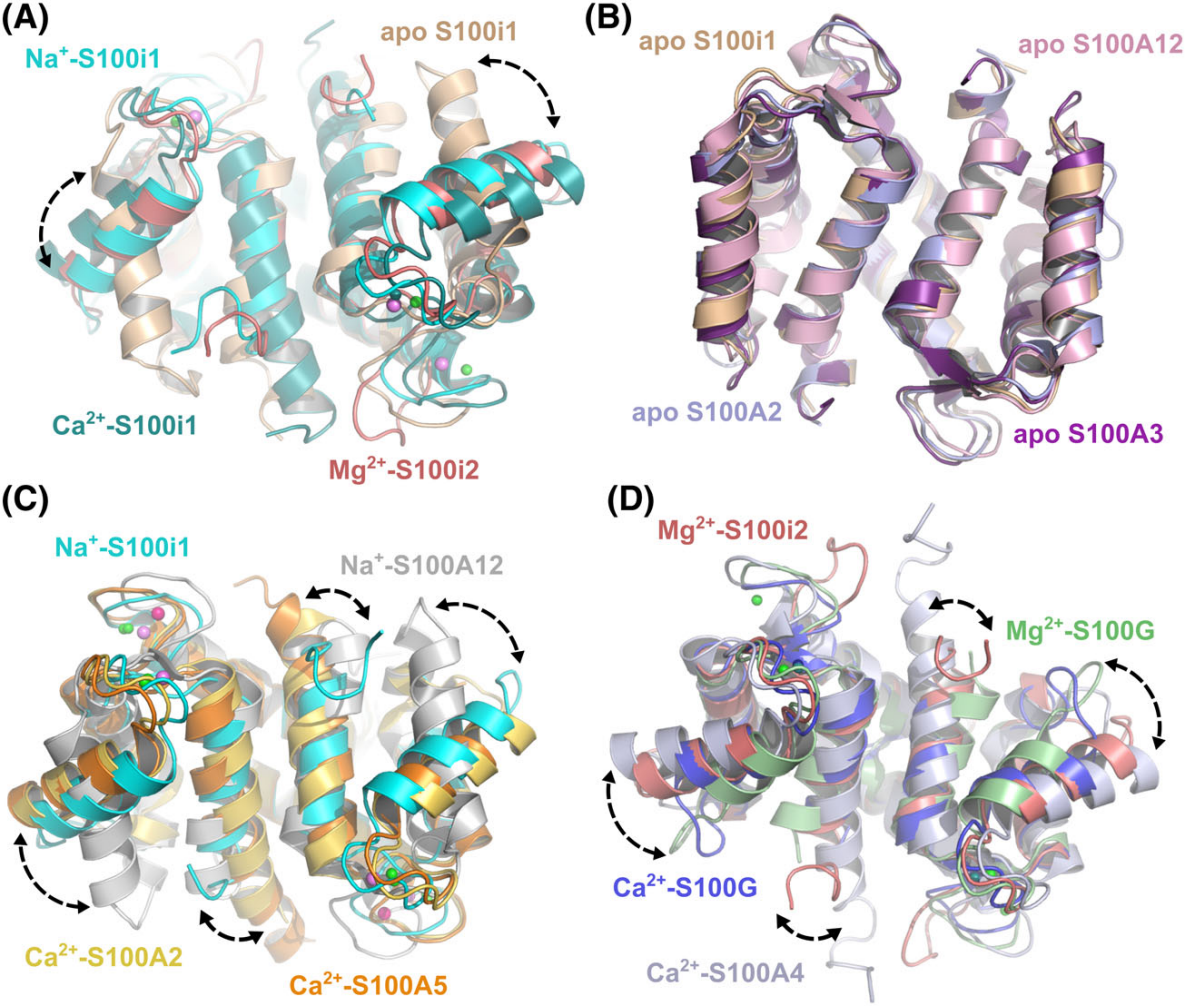
S100i1 and S100i2 undergo conformational rearrangements upon cation binding. (A) Superimposition of the structures of apo S100i1 (beige), Na^+^‐S100i1 (cyan), Mg^2+^‐S100i2 (dark salmon), and AF‐predicted Ca^2+^‐bound S100i1 (teal) homodimers highlights major conformational rearrangements upon occupation of the EF‐loops by Na^+^ or Mg^2+^. The most prominent one is the reorientation of helix H3 in the ion‐bound forms. (B) Comparison of the overall conformation of apo S100i1 with those of apo human S100A2 (light blue, PDB_ID 2RGI [26]), apo human S100A3 (purple, PDB_ID 1KSO [27]), and apo human S100A12 (light pink, PDB_ID 2WCF [28]), highlighting that the protein is in a genuine apo conformation in the absence of cation bound in the EF‐loops. (C) Comparison of the overall conformation of Na^+^‐S100i1 with those of Ca^2+^‐bound human S100A2 (yellow, PDB_ID 4DUQ [29]), Ca^2+^‐bound human S100A5 (orange, PDB_ID 6WN7 [30]) and Na^+^‐bound human S100A12 (gray, PDB_ID 2WCE [28]). Na^+^‐S100i1 adopts a conformation closer to that of Ca^2+^‐bound S100s, in contrast to Na^+^‐S100A12 which resembles more closely the apo conformation of S100 proteins. (D) Comparison of the overall conformation of Mg^2+^‐S100i2 with those of Mg^2+^‐bound human S100G (green, PDB_ID 1IG5 [31]), Ca^2+^‐bound human S100G (blue, PDB_ID 2BCA [32]), and Ca^2+^‐bound human S100A4 (light blue, PDB_ID 2Q91 [16]). Mg^2+^‐S100i2 adopts an intermediary conformation between the fully activated Ca^2+^‐S100A4 one, and the partially activated Mg^2+^‐S100G one, likewise Ca^2+^‐S100G. Structural models were drawn with pymol Molecular Graphics System (version 215 0.99rc6, DeLano Scientific LLC).

Superimposition of our three ictacalcin structures reveals strong conformational movements between the apo form and the ion‐bound models (Fig. 7A). Most notably, H3 undergoes a large rotation upon ion binding, concomitantly with the reorganization of the EF2‐loop around the bound ion. This translates into a large change in the H2–H3 and H3–H4 interhelical angles that shift from 28° and 20°, to 70° and 55°, respectively, from apo to Na^+^/Mg^2+^‐bound states (Table 3). The shift is predicted to be even more drastic in the Ca^2+^‐bound state, as these two interhelical angles switch to 83° and 77°, respectively, in both AF‐models of Ca^2+^‐bound S100i1 and S100i2 homodimers (Fig. 7A; Table 3). The structure of apo S100i1 superimposes best with those of apo S100A12, apo S100A2, and apo S100A3 (Fig. 7B), with root mean square deviation (rmsd) on Cα atoms for the whole dimers of 1.49, 1.53, and 1.70 Å, respectively. The interhelical angles are also well in agreement with those observed for apo S100 models, notably for the H3–H4 angle which fluctuates between 20° and 25° in apo S100 structures (Table 3), thus confirming that this S100i1 structure represents a genuine apo state of the protein.

**Table 3:** Interhelical angles between the different helices of S100i1 and S100i2 according to the cationic state and comparison with the closest human S100 proteins. The angles were calculated using ucsf chimera [33]. The PDB ID of the analyzed structural models is indicated in parentheses.

Protein	H1–H2 angle (°)	H2–H3 angle (°)	H3–H4 angle (°)
Apo S100i1 (this study)	48.2	27.8	20.4
Na^+^‐S100i1 (this study)	45.3	70.6	55.4
Mg^2+^‐S100i2 (this study)	51.5	69.2	56.0
Ca^2+^‐S100i1 (AF model)	40.6	83.0	77.5
Ca^2+^‐S100i2 (AF model)	40.7	82.8	76.9
Apo S100A2 (2RGI)	48.3	35.4	24.0
Apo S100A3 (1KSO)	44.4	43.0	26.2
Apo S100A12 (2WCF)	50.8	37.7	20.3
Na^+^‐S100A12 (2WCE)	51.7	34.5	19.1
Ca^2+^‐S100A2 (4DUQ)	43.1	65.9	64.3
Ca^2+^‐S100A4 (2Q91)	39.4	73.8	71.5
Ca^2+^‐S100A5 (6WN7)	39.4	66.9	68.4
Ca^2+^‐S100G (2BCA)	40.4	72.9	55.5
Mg^2+^‐S100G (1IG5)	47.4	51.5	29.0

In contrast, Na^+^‐bound S100i1 adopts a conformation much closer to that of Ca^2+^‐bound S100A5 and S100A2, except for the positioning of H4 that aligns better with H4 of Na^+^‐bound S100A12 (Fig. 7C). Overall, rmsd on Cα atoms for the whole dimers is 2.05, 2.15 and 3.58 Å, between Na^+^‐bound S100i1 and Ca^2+^‐bound S100A5, Ca^2+^‐bound S100A2, and Na^+^‐bound S100A12, respectively. The interhelical angles for Na^+^‐bound S100i1 are also closer to those observed for Ca^2+^‐bound S100 proteins (Table 3). Importantly, Na^+^‐bound S100A12 only contains one Na^+^ ion, in EF2, whereas our Na^+^‐S100i1 model bears Na^+^ ions in both EF1 and EF2, which may explain the more distant conformational organization between these two structures. Finally, Mg^2+^‐bound S100i2 aligns best with Ca^2+^‐ and Mg^2+^‐bound S100G (Fig. 7D), with rmsd on Cα atoms of 1.45 and 1.48 Å, respectively, and interhelical angles in the same range (Table 3). Interestingly, comparing our S100i2 model with that of Ca^2+^‐bound S100A4 and Ca^2+^‐ or Mg^2+^‐bound S100G highlights the strongest structural similarity with Ca^2+^‐bound S100G, which likewise adopts an intermediary conformation, helices H3 and H4 being positioned half‐way between those of Ca^2+^‐S100A4 and Mg^2+^‐S100G.

Thus, the binding of Na^+^ or Mg^2+^ to S100i1/i2 induces conformational rearrangements within the protein, predominantly driven by the repositioning of helix H3, and yielding intermediary conformations as compared to apo and Ca^2+^‐loaded human S100 structures. Our findings corroborate a recent study from the Harms group where they evidenced calcium‐induced changes in the secondary and tertiary structure of zebrafish S100i1 upon Ca^2+^ addition, using far‐UV circular dichroism and intrinsic fluorescence [12]. These observations demonstrate that S100i1/i2 do not act as calcium buffers, but instead undergo calcium‐dependent conformational activation, like their mammalian counterparts, that may serve as a switch on for their interaction with target effectors.

### The spatiotemporal distribution of ictacalcin transcripts strongly parallels that of mammalian *s100* genes

**Fig. 8:**
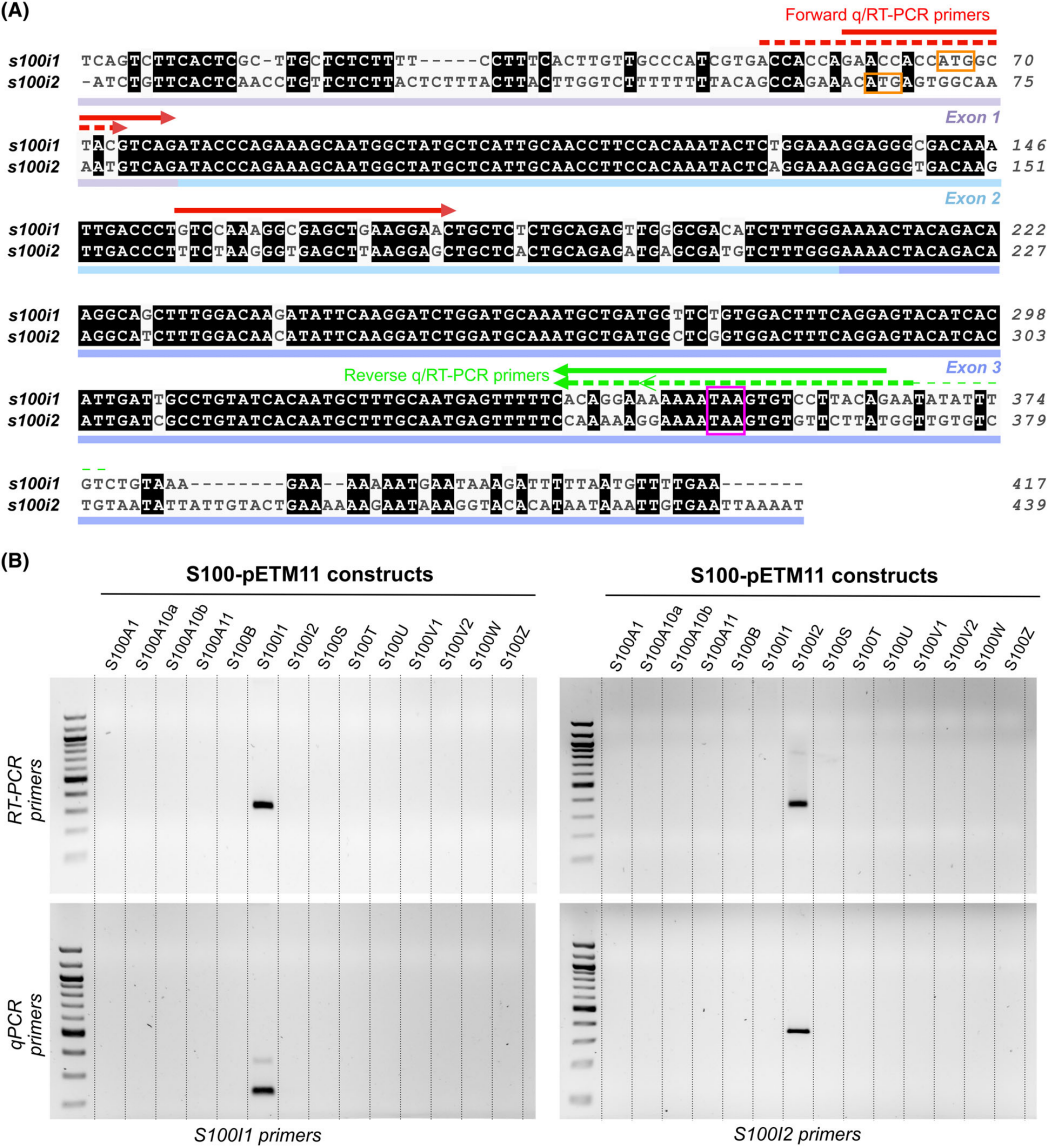
Specificity check for *s100i1*/*i2* RT/qPCR primers. (A) Alignment of the full‐length coding sequences of zebrafish *s100i1* and *s100i2* (NCBI Genbank accession number NM_212761.2 and NM_001082957.2, respectively), done in clustal omega [19] and colored according to sequence conservation with aline [20]. Positions of the three exons are indicated below the sequences. The start and stop codons are marked by orange and purple boxes, respectively. The forward and reverse primers used for semi‐quantitative PCR (RT‐PCR) and quantitative (qPCR) are indicated above the alignment (full line for *s100i1*, dotted line for *s100i2*; see Table S1). (B) Specificity check on the S100‐pETM11 constructs encompassing the 14 distinct *s100* coding sequences. The set of primers chosen for each ictacalcin gene only amplifies the corresponding construct, demonstrating its high specificity. DNA electrophoresis has been performed at least three times on 3 independent PCR reactions (*n* = 3). The DNA ladder employed is the 100 bp DNA ladder from New England Biolabs (product code N3231L).

To further evaluate the resemblance between zebrafish S100i1/i2 and mammalian S100s, we analyzed their *in vivo* spatiotemporal distribution, initially under homeostatic conditions. Previous studies already reported partial expression data for *s100i1*/*i2* genes, using either RT‐qPCR analyses or *in situ* hybridization [8]. The major bottleneck of these investigations, however, lies in the sets of primers and RNA probes employed, which could not distinguish between *s100i1* and *s100i2*, thus providing global data for *i1*/*i2* expression. To circumvent this, we took great care in designing highly specific sets of primers, choosing them in the regions of most divergence between *s100i1* and *s100i2* coding sequences (Fig. 8A). RT‐PCRs performed on plasmids encompassing the 14 different zebrafish *s100* coding DNA sequences confirmed that our primers are highly specific for the target gene and do not amplify the other duplicate gene nor any of the 12 other zebrafish *s100* genes (Fig. 8B).

**Fig. 9:**
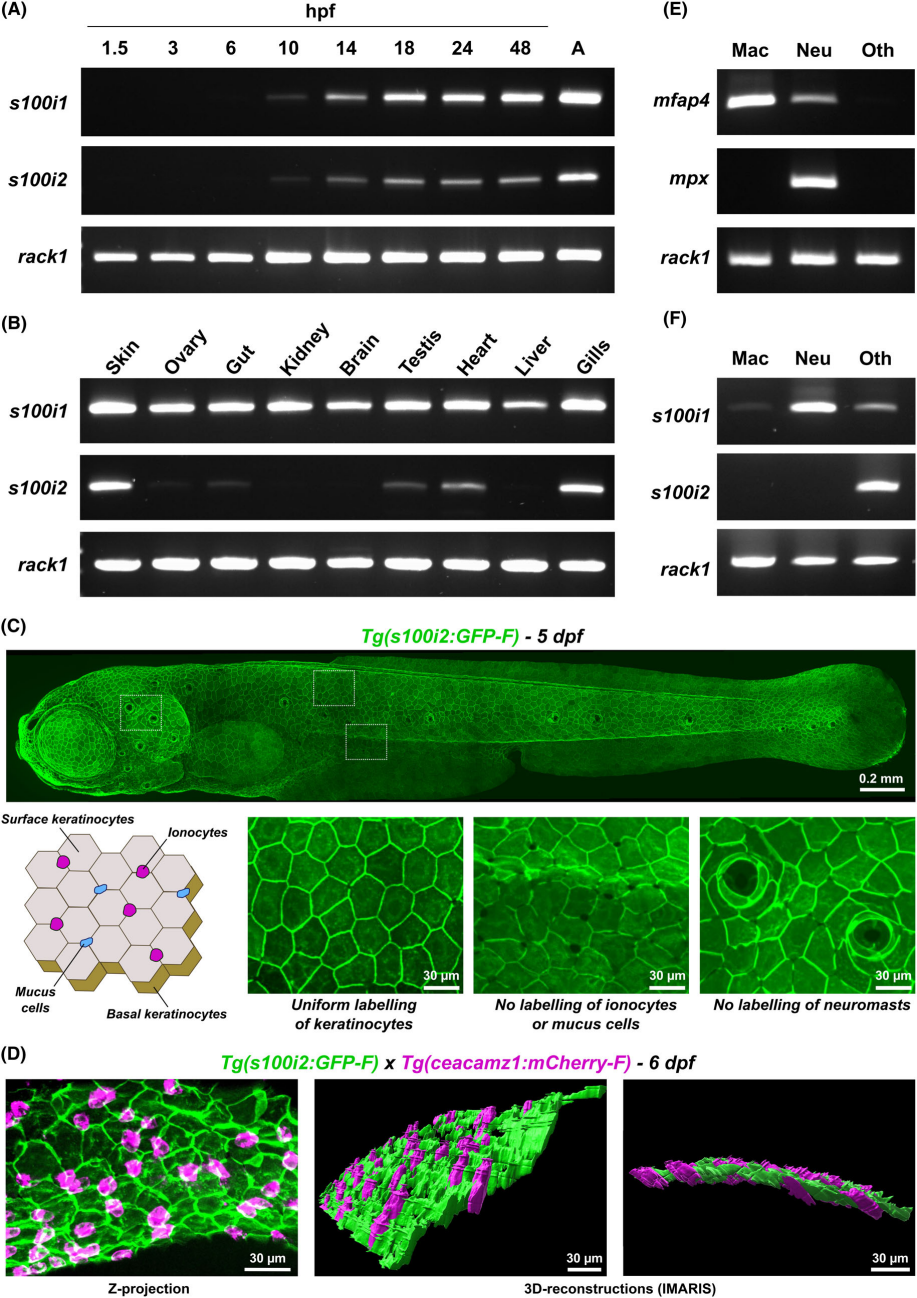
Spatio‐temporal distribution of *s100i1*/*i2* in zebrafish adults and larvae. (A) Representative RT‐PCR analysis of *s100i1/i2* expression during development (developmental stages indicated in hpf). ‘A’ refers to the adult stage (3 months old). Both genes are readily detected at 10 h post fertilization (hpf). (B) Representative RT‐PCR analysis of *s100i1/i2* expression in various tissues of adult zebrafish, highlighting the distinct expression pattern of the two duplicate genes. (C) Live imaging of a 5 days post fertilization (dpf) Tg(*s100i2:GFP‐F*) zebrafish larva using spinning disk confocal microscopy. The upper panel shows a mosaic reconstruction of the whole larva using maximum projections from confocal z‐stacks, highlighting the uniform GFP labeling on the whole epidermis. Lower panels display magnifications of different regions of the epidermis, revealing uniform labeling of all keratinocytes whereas intercalating cells like mucus cells, ionocytes or neuromasts remain unlabelled. A schematic representation of the cell composition of zebrafish skin epidermis at early larval stages is included to ease the identification of the different cell types. The scale bar represents 0.2 mm on the upper image and 30 μm on the lower images. (D) Z‐projection of a portion of the ventral skin of a 6 dpf double‐crossed Tg(*s100i2:GFP‐F*) × Tg(*ceacamz1:mCherry‐F*) zebrafish larva (left) and corresponding 3D‐reconstruction using imaris software (right). The scale bars represent 30 μm. The S100i2‐expressing keratinocytes (green) are located at the same level as Ceacamz1‐expressing ionocytes (magenta), thus corresponding to surface keratinocytes. (E) Representative RT‐PCR analyses using *mfap4* and *mpx* specific primers to evaluate the efficiency of the FACS procedure in obtaining macrophage and neutrophil‐specific RNA samples. (F) Representative RT‐PCR analysis of *s100i1/i2* expression in zebrafish myeloid cells. The housekeeping *rack1* gene was used as a control in all RT‐PCR experiments. All RT‐PCR experiments were performed on at least three independent batches of RNA (*n* = 3).

Given these highly specific primers, we first investigated when *s100i1*/*i2* start being expressed in zebrafish embryos, by conducting RT‐PCRs on total RNA samples collected at different developmental stages. As shown in Fig. 9A, both genes are readily detected at 10 h post fertilization (hpf) and their expression remains stable up to adulthood, suggesting that they are required early during development and then throughout life. Absence of detection before 10 hpf indicates no or minimal maternal contribution to their expression. Next, we examined *s100i1*/*i2* distribution in eight representative adult tissues/organs. Interestingly, the two duplicate genes display rather distinct expression patterns, *s100i1* being broadly expressed in all samples investigated, whereas *s100i2* is mainly restricted to skin and gills, and to a lesser extent to heart, gut, testis, and ovaries (Fig. 9B).

Noteworthily, both genes are strongly expressed in the skin, a feature also shared by mammalian *s100* genes. To further specify where in the skin *s100i1*/*i2* are expressed, we set out to generate reporter lines, which only succeeded for *s100i2*. The resulting transgenic line, Tg(*s100i2:GFP‐F*), drives the expression of a farnesylated version of the GFP protein under the control of a 1.5 kb fragment of the *s100i2* promoter region. The GFP signal uniformly labels all keratinocytes from the zebrafish larval epidermis, whereas interstitial cells like ionocytes, mucus cells or neuromasts remain unmarked (Fig. 9C, zoomed images). Although simpler than the human skin, zebrafish epidermis contains two layers of keratinocytes, both at larval and adult stages: the superficial one, in direct contact with the external environment, and the basal one, lining up against the basement membrane and connecting the underlying dermis (Fig. 9C). To identify which layer(s) of keratinocytes strongly express *s100i2*, we crossed our reporter line with the Tg(*ceacamz1:mCherry‐F*) line, which labels superficial HR‐rich ionocytes [34]. As shown in Fig. 9D, there is no overlap of the green and red fluorescence signals in the larval progeny from this double cross, which confirms that ionocytes are not expressing *s100i2*. 3D‐reconstructions using imaris software further show that the GFP‐labeled keratinocytes are located at the same level as HR‐rich ionocytes on zebrafish epidermis, that is, they correspond to the upper layer of keratinocytes (Fig. 9D). Thus, basal keratinocytes do not seem to produce detectable levels of *s100i2*‐driven GFP, at larval stages at least, suggesting that *s100i2* expression is restricted to surface keratinocytes.

Finally, a hallmark of mammalian S100 proteins, especially the ones acting as pro‐inflammatory alarmins, is their abundant expression in myeloid cells. To evaluate the presence of *s100i1*/*i2* transcripts in zebrafish macrophages and neutrophils, the two myeloid cell types most represented at larval stages, we used a protocol established in our laboratory that allows us to obtain high‐quality RNA samples following FACS‐assisted sorting of macrophage and neutrophil populations from dissociated zebrafish larvae [35]. For this purpose, we used double‐fluorescent 5 dpf larvae from the crossing between reporter line Tg(*mfap4.1*:*mCherry‐F*) (specifically labelling macrophages with red fluorescence; [36]) and reporter line Tg(*mpx*:*GFP*) (specifically labelling neutrophils with green fluorescence; [37]). Efficacy of the cell sorting was first validated by RT‐PCR, using primers that amplify markers specific for macrophages (*mfap4* gene) or neutrophils (*mpx* gene). As seen in Fig. 9E, *mfap4* transcripts are highly enriched in the RNA samples extracted from macrophages whereas *mpx* transcripts are only detected in the RNA samples extracted from neutrophils, thus validating our procedure. Evaluation of *s100i1*/*i2* expression in these cell types (Fig. 9F) shows that *s100i1* is abundantly expressed in neutrophils and more faintly detected in macrophages, while *s100i2* is barely detected in any of these myeloid cells—observations in agreement with the single‐cell RNA seq data on expression of these two genes in zebrafish blood cells provided in the BASICz database (https://www.sanger.ac.uk/tool/basicz/) [38].

Altogether, these data reveal that S100i1/i2 also share features with mammalian S100s at the gene expression level, being abundantly expressed in tissues exposed to the environment, especially the skin, and in cell types that are also important reservoirs for S100 proteins in mammals, such as keratinocytes and myeloid cells.

### Regulation of ictacalcin gene expression in disease context

**Fig. 10:**
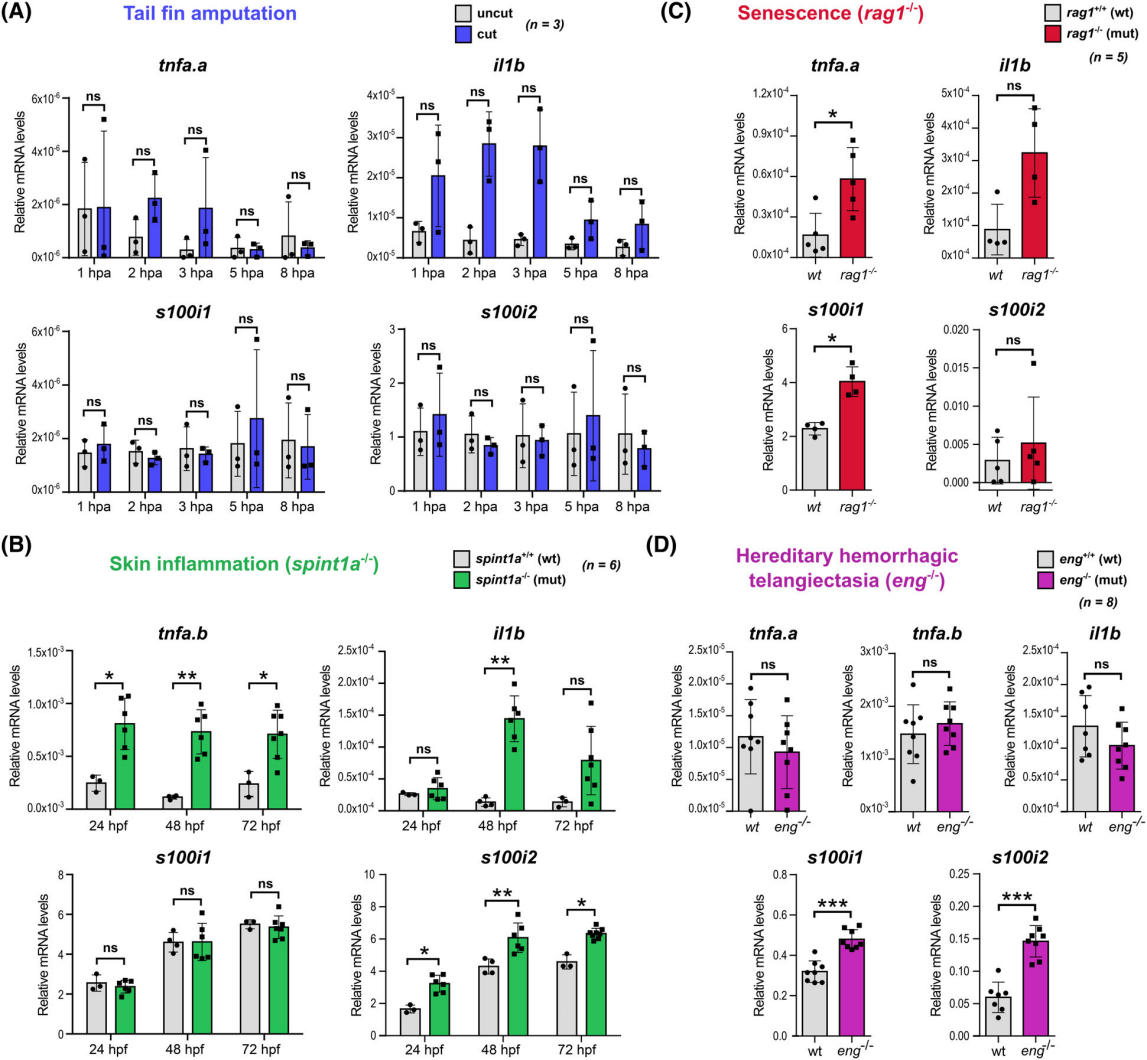
Transcriptional modulation of *s100i1*/*i2* in various zebrafish models of inflammation and/or chronic disease. (A–D) RT‐qPCR analyses of *s100i1/i2* gene expression as compared to the housekeeping gene in the tail fin amputation model in 3 dpf larvae (A), in the *spint1a* ^−/−^ model of skin inflammation in 1 to 3 dpf larvae (B), in the *rag1* ^−/−^ model of senescence in 4 month old adults (C) and in the *eng* ^−/−^ model of Hereditary Hemorrhagic Telangiectasia (HHT) in 1 month old juveniles (D). The relative mRNA levels of the classical pro‐inflammatory markers *tnfa.a*, *tnfa.b* and *il1b* were also evaluated for comparison. The housekeeping *rack1* and *ef1a* genes were used as control in all RT‐qPCR experiments. The number of independent replicates, corresponding to the number of independent batches of larvae for larval models or the number of individual fish for adult models, is as follows: *n* = 3 for the tail fin amputation model (A), *n* = 6 for the *spint1a* ^−/−^ model of skin inflammation (B), *n* = 5 for the *rag1* ^−/−^ model of senescence (C) and *n* = 8 for the *eng* ^−/−^ model of HHT (D). Relative mRNA expression levels are represented as mean ± standard deviation. The statistical significance of the observed differences was analyzed using the non‐parametric Mann–Whitney test. *P*‐values are reported as follows: *: 0.05 > *P* > 0.01; **: 0.01 > *P* > 0.001; ***: 0.001 > *P* > 0.0001; ns: not significant.

In mammals, S100s contribute to many pathologies with deleterious outcomes, as they often undergo upregulation at the transcriptional level, which allows sustaining their pro‐inflammatory action, thereby prompting unresolved inflammation and aggravated tissue damage. To assess whether such transcriptional control also occurs for zebrafish ictacalcins, we investigated their expression levels in representative zebrafish disease models of varying severity. First, we employed the tail fin amputation model on 3 days post fertilization (dpf) larvae, which generates a rapid but rather mild and short‐lived inflammatory insult, as resolution of inflammation initiates within the first day following injury and full regeneration of the amputated fin fold occurs within 3–5 days [39]. In this model, we observe strong transcriptional up‐regulation of pro‐inflammatory cytokine genes such as *tnfa.a* or *il1b* within the first 2–3 h following amputation (hpa), while the levels of mRNA transcripts for these genes rapidly drop down after 5 hpa, indicating the start of the resolution phase (Fig. 10A, upper graphs). In contrast, no significant modulation of *s100i1*/*i2* mRNA levels can be observed in this time window (Fig. 10A, lower graphs).

We next moved to a more intense inflammatory context and investigated the *spint1a* ^−/−^ mutant model, which results in strong skin inflammation with abundant neutrophilic infiltration and a psoriasis‐like phenotype with hyperproliferative keratinocytes [40, 41]. In this model, the inflammatory burden is most intense within the first 3 days of life, healthy phenotype being restored at 5 dpf through gene compensation. Accordingly, we observe strong upregulation of *tnfa.b* and *il1b* mRNA transcripts in mutant larvae at 48 and 72 hpf (as well as 24 hpf for *tnfa.b*) (Fig. 10B, upper graphs). Interestingly, while no modulation of *s100i1* gene expression can be detected, the levels of *s100i2* transcripts are significantly upregulated at all time points investigated in the mutant larvae (Fig. 10B, lower graphs), revealing for the first time transcriptional control of a zebrafish *s100* gene in a larval model of sterile inflammation.

To evaluate whether such modulation may also occur in adult models of sterile inflammation, we used the *rag1* ^−/−^ mutant model of senescence, in which mutant fish lack adaptive immunity and therefore develop early signs of aging, as well as higher innate immune activity [42]. Such a model is highly relevant for S100 biology as mammalian S100s are heavily involved in pathological inflammation in the aged brain [43]. Enhancement of immune activity at the transcriptional level was investigated in the primary lympho‐myeloid organ of the fish, the head kidney, in 4‐month‐old individuals—stage at which the worst inflammatory phenotype is noted. In accordance, both *tnfa.a* and *il1b* transcripts are enriched in the mutant fish at this stage, as compared to wild‐type animals (Fig. 10C, upper graphs). *s100i1* also displays significant transcriptional upregulation while *s100i2* does not show any statistically significant modulation (Fig. 10C, lower graphs).

Finally, we explored *s100i1*/*i2* modulation in the *eng* ^−/−^ mutant model recently developed in our laboratory that recapitulates key symptoms of hereditary hemorrhagic telangiectasia (HHT), a genetic condition causing arteriovenous malformations in humans [44]. It was previously demonstrated that the cardiovascular defects observed in zebrafish *eng* ^−/−^ mutants are associated with chronic hypoxia. On the other hand, the inflammatory status in *eng* ^−/−^ fish has not been assessed yet. Therefore, we first evaluated mRNA transcript levels for the control markers *tnfa* (both *tnfa.a* and *tnfa.b* genes) and *il1b* in 1‐month‐old whole zebrafish. As shown in Fig. 10D (upper graphs), none of these pro‐inflammatory genes is significantly modulated in *eng* ^−/−^ individuals as compared to WT fish. In contrast, both *s100i1* and *s100i2* are significantly upregulated in *eng* ^−/−^ mutants (Fig. 10D, lower graphs).

Altogether, these data demonstrate that teleost‐specific *s100* genes undergo transcriptional up‐regulation in disease contexts associated with sustained inflammation or chronic hypoxia, likewise mammalian S100s.

## Discussion

We here report the very first and detailed characterization of two teleost‐specific S100 proteins, ictacalcins S100i1 and S100i2 from *Danio rerio*, demonstrating that they share all structural features of mammalian S100s, including the two nonequivalent EF‐loop motifs per protomer, the quaternary organization into homodimers, the calcium‐binding capacity, and the calcium‐dependent conformational activation. On the other hand, they do not bind to zinc, suggesting that they do not possess direct antimicrobial activity. This was recently confirmed for S100i1, which failed to restrict bacterial growth of *Staphylococcus epidermidis* and zebrafish‐derived *Aeromonas* or *Vibrio* strains [12]. Nevertheless, many zebrafish *s100* genes, including *s100i1* and *s100i2*, are strongly upregulated following infection by viral or bacterial fish pathogens [45, 46, 47], and in other teleost species, ictacalcin genes are also significantly up‐regulated, both at the gene and protein levels, on pathogen‐exposed mucosal surfaces [48]. Furthermore, our results highlight the strong expression of ictacalcins in tissues and cells with immune functionalities, including skin, gills and neutrophil populations. This parallels the behavior of mammalian S100s, as the most active contributors to innate immunity in humans, S100A7 and the calgranulins S100A8/A9/A12 share similar tissue and cell distribution, being predominantly expressed in the respiratory and digestive tracts (A7/A8/A9), in the skin (A7/A8/A9) and in the bone marrow and lymphoid tissues (all four) [49], as well as in neutrophils where S100A8/A9 constitutes up to 45% of the pool of cytosolic proteins [50]. Altogether, these similarities are good indicators that although not bactericidal *per se*, ictacalcins may contribute to antimicrobial defenses through indirect mechanisms, for instance by modulating immune and inflammatory responses against infectious agents. In addition, their early and abundant presence at embryonic stage suggests that S100i1/i2 may be readily required for protective function in the developing embryo, even before the appearance of functional innate immune cells.

The link we establish here between S100 proteins and zebrafish skin physiology is also interesting. Human skin epidermis constitutes an important reservoir for S100 proteins, as 16 distinct S100s are found in this tissue, contributing to various functions such as epidermal differentiation, keratinocyte proliferation, cell trafficking, or maintenance of mucosal homeostasis, depending on the layer they occupy [51]. Their genes are all clustered together in what is known as the Epidermal Differentiation Complex (EDC), which also encodes other gene families essential for normal epidermal differentiation [51, 52]. Our study demonstrates that zebrafish skin also expresses at least two S100 members. Although their synteny is not conserved with that of mammalian *S100* genes, *s100i1* and *s100i2* cluster with other *s100* genes (*s100a10b*, *s100w*) on zebrafish chromosome 16, as well as with keratinocyte‐associated genes (*krtcap2*) or genes involved in signaling during epidermal differentiation (*efna1b*, *efna3b*), which suggests that S100i1/i2 may also contribute to keratinocyte biology and/or epidermal development. The predominant expression of S100i2 in all epidermal keratinocytes from the upper layer, similar to that already reported for S100i1 [14], is further in favor of conserved functions in mucosal immunity, knowing that these surface keratinocytes also contain high amounts of pro‐inflammatory cytokines such as Il‐1b at larval stages [34, 53].

Another hallmark of mammalian S100s, linked to their alarmin function, is their transcriptional upregulation following prolonged injury, meant to sustain continuous feeding of the pro‐inflammatory response. While up‐regulation of zebrafish *s100* genes was previously demonstrated in several adult models of infection, we here provide the first examples of transcriptional control of teleost‐specific *s100* genes in sterile disease contexts, both in adults and larvae. Importantly, we show that injury models associated with short‐lived and mildly intense inflammatory burden, like the tail fin amputation model, do not elicit transcriptional modulation of *s100i1/i2*. In contrast, more sustained inflammatory damage, like in the *spint1a* ^−/−^ or *rag1* ^−/−^ models, promotes their differential expression. Interestingly, the two ictacalcin genes display distinct modulation in these models. Keratinocyte‐abundant *s100i2* is upregulated in the *spint1a* ^−/−^ model, linked to abnormal keratinocyte behavior, whereas broadly expressed *s100i1* is upregulated in the head kidney of *rag1* ^−/−^ mutants. Differential tissue distribution and transcriptional modulation of *s100i1*/*i2* thus provide another example of two duplicate zebrafish genes that evolved independently following the third whole genome duplication event [54], yielding gene products that may exert distinct biological roles rather than function as redundant proteins.

Our evidence that *s100i1*/*i2* gets transcriptionally modulated during long‐lasting inflammatory insult again establishes a strong parallel with mammalian S100s and hints at a possible role of zebrafish ictacalcins in the development of deleterious inflammatory phenotypes. This may be particularly true for skin pathologies in which mammalian S100s are strongly implicated, like skin cancers, dermatitis or psoriasis [55]. For example, keratinocyte‐derived S100A7 and S100A15 are strongly upregulated in human psoriatic skin and drive the chemotactic recruitment of immune cells together with the sustained production of pro‐inflammatory cytokines, further potentiating the inflammatory cascade [56]. Zebrafish *spint1a* ^−/−^ mutant provides a highly relevant model for this pathology, with hyperproliferative behavior of keratinocytes, aberrant immune infiltration and epidermal aggregates mimicking psoriatic lesions [57]. The upregulation of *s100i2* observed in this model, concomitant to that of pro‐inflammatory markers *tnfa.b* and *il1b*, thus strengthens the parallel with the human pathology and calls for a deeper evaluation of the role possibly played by zebrafish ictacalcins in the propagation of the psoriatic inflammatory symptoms.

Finally, we also evidenced transcriptional upregulation of *s100i1*/*i2* in the *eng* ^−/−^ model, which may not be characterized by an underlying inflammatory context, since we did not detect significant upregulation of classical pro‐inflammatory markers. This model is associated with chronic hypoxia [44], a condition often encountered in a cancer context in humans and to which S100 proteins have already been shown to respond and/or contribute [58], either through hypoxia‐driven up‐regulation of *S100* genes [59, 60] or through S100‐dependent modulation of HIF‐1α expression [61]. Many of these processes rely on the receptor for advanced glycation end‐products (RAGE), which is absent in zebrafish. A direct implication of S100i1/i2 in modulating hypoxic conditions in the *eng* ^−/−^ model—and more generally in disease contexts in zebrafish—thus remains an open question, but other S100 receptors highly conserved in zebrafish, such as Toll‐like receptors or yet‐to‐discover teleost‐specific S100 receptors, may relay such function.

In conclusion, our study highlights the strong similarities existing between teleost‐specific S100i1/i2 and mammalian S100s, despite their distant phylogenetic lineage. Such similarities hint at a conserved role in regulating immune and inflammatory responses in chronic disease contexts. Defining the exact nature and extent of this role will require further investigation, including the generation of KO lines or loss‐of‐function mutants to be used in the zebrafish inflammatory models we have already explored.

## Materials and methods

### Expression and purification of recombinant S100i1 and S100i2 from *Danio rerio*

The full‐length ORFs of zebrafish S100i1 and S100i2 proteins were PCR‐amplified from *Danio rerio* genomic DNA and cloned in frame after the sequence coding for Tobacco Etch Virus (TEV) protease cleavage site in pETM11 vector (EMBL vector collection), using restriction‐free cloning [62]. CterW variants of S100i1 and S100i2 were generated from these constructs using PCR‐based site‐directed mutagenesis with High Fidelity Phusion DNA Polymerase (New England Biolabs, Ipswich, MA, USA) and anticomplementary oligonucleotides bearing the Trp insertion. For the expression of S100i1 and S100i2, the pETM11 constructs were transformed into heat‐competent *Escherichia coli* BL21 (DE3) (ThermoFisher Scientific, Waltham, MA, USA). Cells were grown at 37 °C in 2xYT medium supplemented with 50 μg·mL^−1^ of kanamycin, until they reached an exponential phase of growth. Protein expression was induced by adding 1 mm IPTG and further incubating overnight at 18 °C. The recombinant proteins were then purified to homogeneity from *E. coli* soluble cell extracts, using our established 3‐step procedure [63] consisting of a first Nickel‐affinity chromatography (Ni‐NTA), followed by TEV cleavage and counter‐purification on Ni‐NTA, and a final step of size exclusion chromatography (SEC) performed on a 24 mL Superdex 75 Increase 10/300 GL column (Cytiva, Marlborough, MA, USA) equilibrated with 20 mm Tris–HCl pH 7.5, 100 mm NaCl.

### Crystallization and data collection

Purified S100i1/i2 proteins in buffer 20 mm Tris–HCl pH 7.5, 100 mm NaCl were concentrated to 10 mg·mL^−1^ and set to crystallize either in apo form or after supplementation with 5 mm CaCl_2_ (5 eq), using the sitting‐drop vapor diffusion method in 48‐ or 96‐well plates (Swissci, Wycombe, UK) and commercial crystallization screens (Molecular Dimensions Ltd, Rotherham, UK). Crystal forms 1 and 2 of S100i1 appeared at 291K over a reservoir containing 0.2 m Li sulfate, 0.1 m Tris–HCl pH 8.5, 25% PEG 4000 or 0.2 m Na acetate, 0.1 m Tris–HCl pH 9.5, 35% PEG 4000, respectively, while S100i2 crystals grew at 291K using 0.2 m MgCl_2_, 0.1 m Tris HCl pH 8.5, 30% PEG 4000 as the crystallization solution.

Prior to data collection, S100i1 crystals were soaked in a cryoprotective solution corresponding to the crystallization condition supplemented with 10% (crystal form 1) or 20% (crystal form 2) glycerol before flash‐freezing in liquid nitrogen. S100i2 was directly flash‐frozen in liquid nitrogen. Complete X‐ray diffraction datasets were collected at a wavelength of 1.0 Å on the X06DA beamline at the Swiss Light Source facility (SLS, Switzerland).

### Structure determination and refinement

Datasets were processed with xds [64]. Both crystal forms of S100i1 belonged to space group P2_1_ while S100i2 crystallized in the P2 space group. The structures of Na^+^‐S100i1 and Mg^2+^‐S100i2 were determined by molecular replacement (MR) using an automated model search with Balbes [65]. Interestingly, although S100i1 and S100i2 share 87% sequence identity, the structural models from two distinct S100 proteins were used for the MR search, namely that of human Ca^2+^‐bound S100A2 [29] for S100i1 and that of human Ca^2+^‐bound S100A4 [16] for S100i2. The resulting S100i1 model was then employed to phase the X‐ray data from apo S100i1, using MR in phaser [66]. All three models were refined by alternating cycles of manual rebuilding in coot [67] and cycles of energy minimization in phenix.refine [68], including refinement of individual isotropic Atomic Displacement Parameters (ADP) and Translation–Libration–Screw (TLS) parameterization (Table 1). S100i1 crystal form 2 and S100i2 crystals showed strong translational non‐crystallographic symmetry (tNCS), which yielded final *R* and *R*_free_ values much higher than expected for the resolution. Nevertheless, the electron density maps were overall well defined and allowed for the accurate rebuilding of all three structural models, except in a few loop regions showing more flexibility. The quality of the final models was assessed with molprobity [69].

### Sequence and structural analyses

Sequence alignments were done in clustal omega [19] and coloring according to conservation was done in aline [20]. Analysis of S100 dimeric assemblies and interfaces was performed in pisa [15]. Search for the closest structural homologs was done with pdbefold [70]. Calculations of interhelical angles were performed with ucsf chimera, developed by the Resource for Biocomputing, Visualization, and Informatics at the University of California, San Francisco, with support from NIH P41‐GM103311 [33]. 3D‐modeling of Ca^2+^‐bound S100i1 and S100i2 homodimers was performed with the alphafold3 server [71]. Structural alignments and rmsd calculations were made with the pymol Molecular Graphics System (version 215 0.99rc6, DeLano Scientific LLC, San Francisco, CA, USA). All figures were drawn in pymol.

### Isothermal titration calorimetry (ITC)

For ITC experiments, S100i1 and S100i2 proteins were purified as stated above except that they were run on Chelex^®^ resin prior to SEC, to remove all traces of cations, and the last step on SEC was performed either in Buffer 1 (20 mm Tris pH 7.5, 100 mm NaCl; for calcium titrations) or in Buffer 2 (20 mm Tris pH 7.5, 100 mm NaCl, 2 mm CaCl_2_; for zinc titrations). Frozen proteins were thawed on ice before use. ITC experiments were performed in a MicroCal PEAQ‐ITC (Malvern Panalytical Ltd, Malvern, UK) at 25 °C. The first injections were 0.4 μL, followed by 19 × 2 μL injections with constant stirring. To achieve Ca^2+^ binding saturation, a second series of injections was performed and the resulting ITC data files were concatenated into a single data file for analysis. S100i1 and S100i2 were used at 40 and 42–60 μm in the ITC cell, respectively; 0.4–0.8 mm CaCl_2_ or 0.6–2.0 mm Zn acetate solutions were used in the syringe. ITC data were integrated and baseline corrected using nitpic [22]. The integrated data were globally analyzed in sedphat [72] using a model with two symmetric binding sites (macroscopic *K*_D_) or two non‐symmetric sites (microscopic *K*_D_). We also included a floating ‘fraction incompetent’ parameter to capture uncertainty in the relative protein and Ca^2+^ concentrations. Thermogram and binding figures were plotted in gussi [73].

### Zebrafish lines, maintenance and ethics

All zebrafish experiments described in the present study were conducted by authorized staff in compliance with the European Union guidelines for handling of laboratory animals (2010/63/EU Directive) and the ARRIVE guidelines [74]. Breeding and maintenance of adult fish were performed at the ZEFIX‐LPHI (CNRS, University of Montpellier, Montpellier, France; license number CEEA‐LR‐B34‐172‐37) and University of Murcia (CARM approval number #A13220914) fish facilities, according to the local animal welfare standards approved by the Direction Sanitaire et Vétérinaire de l'Hérault, the Comité d'Ethique pour l'Expérimentation Animale of Languedoc‐Roussillon, the French Ministère de l'Enseignement Supérieur de la Recherche et de l'Innovation under reference APAFIS #36309‐2022040114222432 V2 and by Comunidad Autónoma de la Región de Murcia, Dirección General de Ganadería, Pesca y Acuicultura.

Fish and embryo maintenance, staging and husbandry were as described previously [34]. Experiments were performed using wild‐type AB, *slc24a5* ^*b1/b1*^ mutant (*golden* [75]) or *mitfa* ^*w2/w2*^ *mpv17* ^*a9/a9*^ double‐mutant (*casper* [76]) zebrafish strains from Zebrafish International Resource Center (ZIRC) along with the following transgenic lines we generated: Tg(*mfap4.1:mCherry‐F*) ump6Tg to label macrophages [36], Tg(*mpx:GFP*) i114Tg to label neutrophils [37], Tg(*ceacamz1:mCherry‐F*) ump9Tg to label epidermal HR‐ionocytes [34], Tg(*s100i2:GFP‐F*) ump16Tg (this study) to visualize the transcriptional expression of *s100i2*, and the following mutant lines corresponding to loss‐of‐function for the targeted genes: *spint1a* ^hi2217Tg/hi2217Tg^ or *spint1a* ^−/−^ (Serine Peptidase Inhibitor, Kunitz Type 1a gene) [40], *eng* ^−/−^ (Endoglin gene) [44] and *rag1* ^*t26683/t26683*^ or *rag1* ^−/−^ (Recombination activating gene 1) [42, 77]. Zebrafish embryos were obtained from pairs of adult fish by natural spawning and raised at 28 °C under a 14 : 10‐h light/dark cycle. All experimentations on live larvae were performed under anesthesia with tricaine (ethyl 3‐aminobenzoate). When required, euthanasia was achieved using an overdose of tricaine (500 mg·L^−1^).

### FACS‐sorting of macrophages and neutrophils

Macrophage and neutrophil cell populations were sorted from dissociated zebrafish larvae using FACS as previously documented [35]. Briefly, for one batch, 120 larvae obtained from the crossing between Tg(*mfap4.1:mCherry‐F*) and Tg(*mpx:GFP*) lines, and selected based on double‐positive fluorescence, were dissociated at 5 or 6 dpf using our established enzyme‐free cell dissociation procedure. After final resuspension in buffer 0.9× Dulbecco's Phosphate‐Buffered Saline (DPBS) supplemented with 2% heat‐inactivated Fetal Bovine Serum (FBSi) and 2 mm EDTA, SYTOX™ Red Dead Cell Stain was added in the sample (5 nm final concentration) to label nonviable cells. Live mCherry‐positive, GFP‐positive and double‐negative cell populations were then sorted by FACS on a BD FACSAria™ IIu cell sorter on the Montpellier RIO Imaging microscopy platform (MRI, Biocampus, Montpellier), using gating strategy and fluorescence detection parameters as previously described [35]. Sorted cells were directly collected into RNase/DNase‐free 1.5‐mL microfuge tubes containing 100 μL of lysis buffer from the QIAGEN RNeasy Micro kit (RLT buffer supplemented with 1% v/v β‐mercaptoethanol) (QIAGEN, Hilden, Germany). The samples were vigorously vortexed and then immediately flash‐frozen in liquid nitrogen for storage at −80 °C until proceeding with RNA extraction.

### Total RNA extraction

For *s100i1*/*i2* expression studies at larval stages, 25–50 embryos/larvae from the desired zebrafish lines were pooled and their total RNA was isolated with the NucleoSpin RNA Plus kit (Macherey‐Nagel, Düren, Germany). Expression studies during development were performed on dechorionated WT AB larvae collected at various developmental stages (1.5–48 hpf). Total RNA for adult control was extracted from 2 to 3 adult fish (3 months old) using the NucleoZOL protocol (Macherey‐Nagel). Expression studies in the tail fin amputation and *spint1a* ^−/−^ models were conducted on tail‐fin amputated versus intact 3 dpf WT AB larvae or on *spint1a* ^−/−^ versus *casper* (*spint1a* ^+/+^) 24/48/72 hpf larvae, respectively. RNA samples for expression studies in adult tissues/organs were obtained previously [34]. RNA samples for expression studies in *rag1* ^−/−^ (head kidney of 4 months old zebrafish), *eng* ^−/−^ (1 month old whole fish) and wild‐type siblings (*rag1* ^+/+^, *casper* background; *eng* ^+/+^, AB background) were from previous reports [42, 44]. Macrophage‐specific, neutrophil‐specific and double‐negative total RNAs were extracted from FACS‐sorted samples using the RNeasy Micro kit (QIAGEN) and RNA sample quality and concentration were assessed using the RNA 6000 Pico kit (Agilent, Santa Clara, CA, USA) on an Agilent 2100 Bioanalyzer System (qPhD platform, Montpellier). All expression studies were performed on at least three independent replicate batches, for each time point/stage/condition investigated.

### Semi‐quantitative and quantitative PCR

For primer specificity check, the full‐length coding sequences of all 14 zebrafish *s100* genes were amplified from *D. rerio* cDNA and cloned into pETM11 vector using RF‐cloning. For expression studies, equal amounts of total RNAs (500 ng) were reverse‐transcribed using Oligo(dT) primers and M‐MLV reverse transcriptase (ThermoFisher Scientific). For RNA samples extracted from FACS‐sorted cells, only 4 ng of total RNAs were used for reverse transcription due to low yields of RNA. Semi‐quantitative PCR analyses (RT‐PCRs) were performed with an amount of cDNA equivalent to 5 ng total RNA or 0.1 ng total RNA for expression studies in myeloid cells or on 10 ng of purified pETM11‐S100 plasmid, using High Fidelity Phusion DNA polymerase (New England Biolabs) and specific sets of primers for each gene as listed in Table S1.

Quantitative PCRs (qPCRs) were carried out using an amount of cDNA equivalent to 0.5 ng total RNA in SYBR® Green mix (SensiFast™ SYBR^®^, Meridian BioScience, Cincinnati, Ohio, USA) in the presence of 600 nm of each primer (as indicated in Table S1). Reaction mixes were assembled in 384‐well plates with the help of a Labcyte Echo 525 Liquid Handler and qPCRs were run in triplicates using a LightCycler^®^ 480 II thermal cycler (Roche, Basel, Switzerland) available at the Montpellier GenomiX's High‐throughput qPCR facility (MGX, Biocampus, Montpellier). Data were analyzed with the lc480 software and results are presented as relative target gene mRNA expression as compared to the reference gene (*rack1* or *ef1a*).

### Generation of the Tg(*s100i2:GFP‐F*) fluorescent reporter line

The Tg(*s100i2:GFP‐F*) transgenic reporter line was generated following previously reported procedures [34, 53]. A 1.5 kb fragment of the *icn2* (*s100i2*) promoter was amplified from zebrafish genomic DNA using 5′‐CTTCAAAATGACAATTCCCATACTTG‐3′ and 5′‐CATGTTTCTGGCTGTAAAAAAAAGAC‐3′ as forward and reverse primers. This fragment, which terminates at the *icn2* AUG start codon, was inserted just upstream of the reading frame of farnesylated GFP protein (GFP‐F) in the I2BN‐modeGFP transgenesis vector, using restriction‐free cloning. This vector, derived from pBluescript, harbors two I‐SceI homing sites [78]. The resulting plasmid was injected into one‐cell stage embryos (wild‐type AB zebrafish line) together with the I‐SceI meganuclease. F0‐microinjected embryos were screened for transgene expression at 1 and 2 dpf, and a stable line was established. The line has been registered as ump16Tg in ZFIN.

### Confocal microscopy and image analysis

Imaging of 5 and 6 dpf larvae from the Tg(*s100i2:GFP‐F*) reporter line or from double‐cross with the Tg(*ceacamz1:mCherry‐F*) line was done by confocal microscopy. Prior to live imaging, larvae were anesthetised with 0.016% (w/v) tricaine and immobilized in 1% low‐melting point agarose in 35 mm glass‐bottom FluoroDish plates (World Precision Instruments, Hitchin, UK). Fluorescence image stacks were acquired at 28 °C using a spinning disk Nikon Ti Andor CSU‐W1 confocal microscope mounted with 20×/0.75 or 40×/1.15 Water objectives and an ANDOR Neo sCMOS camera (lasers 488 nm for GFP and 561 nm for mCherry). Images were processed with fiji (image j software) and compressed into maximum intensity Z‐projections. 3D‐reconstructions were generated with imaris software (Oxford Instruments, Abingdon, UK. https://imaris.oxinst.com). Image acquisition and processing were performed on the Montpellier RIO Imaging microscopy platform (MRI, Biocampus, Montpellier).

### Statistical analyses

Expression studies were designed to generate experimental groups of comparable size, defined from preliminary experiments. No inclusion/exclusion criteria of data were applied. Relative mRNA expression levels were represented as mean ± standard deviation. graphpad prism v8.3.0 (San Diego, CA, USA) software was used to construct graphs and analyze data. The nonparametric Mann–Whitney test was employed for statistical analyses and the *P*‐value < 0.05 was considered as the threshold for the statistical significance of differences between groups. The number of independent experiments and the *P*‐value definitions are indicated in the figure legends where appropriate.

## Conflict of interest

The authors declare no conflict of interest.

## Author contributions

LY designed the study; LY, MN‐C, VM, EL, and SDT planned the experiments; LH, TP, MD, CB‐P, JFRV, SDT, CBi, CBu, CG, JG, EL, and LY performed experiments; LY, MN‐C, VM, EL, and SDT provided student and technical staff supervision; LH, TP, MD, CBi, and LY analyzed the data and prepared the figures; LY wrote the original manuscript draft of the paper; all authors discussed the results and contributed to the final version of the manuscript.

## Supporting information


**Table S1.** List of primers used in this study for semi‐quantitative and quantitative RT‐PCRs.

## Data Availability

Atomic coordinates and structure factors for the three crystallographic structures reported in this article have been deposited in the Protein Data Bank under accession codes 9IN2 (apo S100i1), 9HYG (Na^+^‐bound S100i1) and 9I1G (Mg^2+^‐bound S100i2). The AF‐predicted models of Ca^2+^‐bound S100i1 and S100i2 homodimers are available in ModelArchive (https://www.modelarchive.org; [79]) with the accession codes ma‐bku33 and ma‐iqag2 for S100i1 and S100i2, respectively.
