# Supplementary material for: Teleost‐specific ictacalcins exhibit similar structural organization, cation‐dependent activation, and transcriptional regulation as human S100 proteins
Source: FEBS J. 2025 Nov 28;293(7):2042–66. doi: 10.1111/febs.70354 (PMC13044992; doi:10.1111/febs.70354)
Supplement: Supplementary file 1 — Table S1. List of primers used in this study for semi‐quantitative and quantitative RT‐PCRs. [file FEBS-293-2042-s001.pdf]

## Supporting Information

# Teleost-specific ictacalcins exhibit similar structural organization, cation-dependent activation and transcriptional regulation as human S100 proteins

*Liz Hernández[1], Théo Paris[1], Maria Demou[1], Catherine Birck[2], Christina Begon-Pescia[1], Juan Francisco Rodríguez Vidal[3,4,5], Sylwia D. Tyrkalska[3,4,5], Charlotte Bureau[1], Catherine Gonzalez[1], Juliette Gracia[1], Etienne Lelièvre[1], Victoriano Mulero[3,4,5], Mai Nguyen-Chi[1], Laure Yatime[1,*]*

[1] LPHI, University of Montpellier, INSERM, CNRS, Montpellier, France

[2] ISB Platform, CBI-IGBMC, CNRS, INSERM, University of Strasbourg, Illkirch, France

[3] Departamento de Biología Celular e Histología, Facultad de Biología, Universidad de Murcia, Spain

[4] Instituto Murciano de Investigación Biosanitaria (IMIB) Pascual Parrilla, Murcia, Spain

[5] Centro de Investigación Biomédica en Red de Enfermedades Raras (CIBERER), Instituto de Salud Carlos III, Madrid, Spain

* Correspondence to: laure.yatime@inserm.fr

**CONTENTS**


| | |
|---|---|
| **Table S1** | List of primers used in this study for semi-quantitative and quantitative RT-PCRs |

| Gene | Primer sequence | Application |
|---|---|---|
| *s100i1* | Forward: 5’-GAACCACCATGGCTACGTCAG-3’<br>Reverse: 5’-CTGTAAGGACACTTATTTTTTTCCTGTG-3’ | *Semi-quantitative RT-PCR* |
| | Forward: 5’-GTCCAAAGGCGAGCTGAAGGAAC-3’<br>Reverse: 5’-CTGTAAGGACACTTATTTTTTTCCTGTG-3’ | *qRT-PCR* |
| *s100i2* | Forward: 5’-GCCAGAAACATGAGTGGCAAAATG-3’<br>Reverse: 5’-CCATAAGAACACACTTATTTTCCTTTTTGG-3’ | *Semi-quantitative RT-PCR* |
| | Forward: 5’-GCCAGAAACATGAGTGGCAAAATG-3’<br>Reverse: 5’-CAGACACAACCATAAGAACACACTTATTTTCC-3’ | *qRT-PCR* |
| *mfap4* | Forward: 5’-GCTGTTGAGGAGAGAGTGAGAAGATG-3’<br>Reverse: 5’-GTCAGCTGGTAGAGGTTCTCTAGTC-3’ | *Semi-quantitative RT-PCR* |
| *mpx* | Forward: 5’-GGCTGCTGTTGTGCTCTTTCAATG-3’<br>Reverse: 5’-GGTTTGAGCTTCACAGCCTGTCATAC-3’ | *Semi-quantitative RT-PCR* |
| *il1b* | Forward: 5’-TGGACTTCGCAGCACAAAATG-3’<br>Reverse: 5’-GTTCACTTCACGCTCTTGGATG-3’ | *qRT-PCR* |
| *tnfa.a* | Forward: 5’-TTCACGCTCCATAAGACCCA-3’<br>Reverse: 5’-CCGTAGGATTCAGAAAAGCG-3’ | *qRT-PCR* |
| *tnfa.b* | Forward: 5’-CGAAGAAGGTCAGAAACCCA-3’<br>Reverse: 5’-GTTGGAATGCCTGATCCACA-3’ | *qRT-PCR* |
| *rack1* | Forward: 5’-CCTCGCCAAAATGACCGAGC-3’<br>Reverse: 5’-GGTGTACTTGCAGACTCCCAG-3’ | *Semi-quantitative RT-PCR* |
| | Forward: 5’- CAGTTTGCTCTGTCTGGATCCTGG-3’<br>Reverse: 5’- GTGTACTTGCAGACTCCCAGAGTG-3’ | *qRT-PCR* |
| *ef1a* | Forward: 5’-TTCTGTTACCTGGCAAAGGG-3’<br>Reverse: 5’-TTCAGTTTGTCCAACACCCA-3’ | *qRT-PCR* |

**Table S1. List of primers used in this study for semi-quantitative and quantitative RT-PCRs.**
